# RNF31 induces paclitaxel resistance by sustaining ALYREF cytoplasmic–nuclear shuttling in human triple‐negative breast cancer

**DOI:** 10.1002/ctm2.70203

**Published:** 2025-02-06

**Authors:** Shumei Huang, Dongni Shi, Shuqin Dai, Xingyu Jiang, Rui Wang, Muwen Yang, Boyu Chen, Xuwei Chen, Lingzhi Kong, Lixin He, Pinwei Deng, Xiangfu Chen, Chuyong Lin, Yue Li, Jun Li, Libing Song, Yawei Shi, Weidong Wei

**Affiliations:** ^1^ Department of Experimental Research State Key Laboratory of Oncology in South China Collaborative Innovation Center for Cancer Medicine Sun Yat‐sen University Cancer Center Guangzhou China; ^2^ Department of Biochemistry Zhongshan School of Medicine Sun Yat‐sen University Guangzhou China; ^3^ Department of Medicinal Laboratory State Key Laboratory of Oncology in South China Collaborative Innovation Center for Cancer Medicine Sun Yat‐sen University Cancer Center Guangzhou China; ^4^ Department of Radiation Oncology Shenzhen Key Laboratory of Gastrointestinal Cancer Translational Research Cancer Institute Peking University Shenzhen Hospital Shenzhen‐Peking University‐Hong Kong University of Science and Technology Medical Center Shenzhen China; ^5^ Department of Breast and Thyroid Surgery the First Affiliated Hospital of Sun Yat‐sen University Guangzhou China; ^6^ Breast Oncology Department State Key Laboratory of Oncology in South China Collaborative Innovation Center for Cancer Medicine Sun Yat‐sen University Cancer Center Guangzhou China

**Keywords:** ALYREF, cytoplasmic–nuclear shuttling, paclitaxel resistance, triple‐negative breast cancer

## Abstract

**Background:**

Resistance to paclitaxel‐based chemotherapy is the major obstacle in triple‐negative breast cancer (TNBC) treatment. However, overcoming paclitaxel resistance remains an unsolved problem. The present study aimed to determine whether paclitaxel treatment impairs Aly/REF export factor (ALYREF) cytoplasmic–nuclear shuttling, its mechanism, and the role of ubiquitinated ALYREF in paclitaxel resistance.

**Methods:**

The subcellular proportion of ALYREF was detected in samples from patients with TNBC using immunohistochemistry to analyze the relationship between ALYREF distribution and paclitaxel response. Cell viability assays, immunofluorescence assays, quantitative real‐time reverse transcription PCR assays, western blotting, and terminal deoxynucleotidyl transferase nick‐end‐labelling assays were conducted to measure the biological function of the subcellular proportion of ALYREF and E3 ligase ring finger protein 31 (RNF31) on paclitaxel sensitivity in TNBC. The synergistic effects of an RNF31 inhibitor plus paclitaxel on TNBC were evaluated. Cox regression models were adopted to assess the prognostic role of RNF31 in TNBC.

**Results:**

Herein, we showed that regulation of ALYREF cytoplasmic–nuclear shuttling is associated with the paclitaxel response in TNBC. In paclitaxel‐sensitive TNBC, ALYREF was trapped in the cytoplasm by paclitaxel, while in paclitaxel‐resistant TNBC, ALYREF was efficiently transported into the nucleus to exert its function, allowing the export of the mRNAs encoding paclitaxel‐resistance‐related factors, including tubulin beta 3 class III (TUBB3), stathmin 1 (STMN1), and microtubule‐associated protein Tau (TAU), ultimately inducing paclitaxel resistance in TNBC. Mechanistically, we found that RNF31 interacts with and ubiquitinates ALYREF, which facilitates ALYREF nuclear transportation via importin 13 (IPO13) under paclitaxel treatment. Notably, the RNF31 inhibitor and paclitaxel synergistically repressed tumour growth in vivo and in TNBC patient‐derived organoids. In addition, analysis of patients with TNBC showed that elevated RNF31 levels correlated with poor prognosis.

**Conclusion:**

These data indicated that RNF31‐mediated ALYREF ubiquitylation could represent a potent target to reverse paclitaxel resistance in TNBC.

**Key points:**

- RNF31 facilitated ALYREF‐mediated PTX resistance in TNBC.
- RNF31 promoted ALYREF nuclear transport via IPO13 in response to PTX treatment, subsequently enhancing the export of mRNAs encoding PTX resistance‐related factors, including TUBB3, STMN1, and TAU.
- Blocking RNF31 trapped ALYREF in the cytoplasm and induced TNBC cell death upon PTX treatment.
- Inhibiting RNF31 activity re‐sensitized PTX‐resistant TNBC to PTX treatment.

## Graphical abstract

An overview of the paclitaxel resistance mechanism induced by RNF31‐mediated ALYREF subcellular distribution. RNF31 promoted the linear ubiquitination of ALYREF, subsequently enhancing ALYREF nuclear transport via IPO13 upon PTX treatment. In addition, RNF31‐mediated ubiquitination of ALYREF facilitated ALYREF binding and export of PTX resistance‐related factors, including TUBB3, STMN1, and TAU, ultimately leading to PTX resistance in TNBC. Thiolutin, an inhibitor of RNF31, enhanced PTX sensitivity in TNBC.

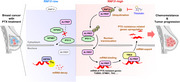

## 1 BACKGROUND

Triple‐negative breast cancer (TNBC), the most aggressive of breast cancer subtypes, accounts for 15 to 20% of all cases of breast cancer.^1^ Considering the negative expression of estrogen receptor (ER), progesterone receptor (PR), and human epidermal growth factor receptor 2 (HER2/ErbB2), chemotherapy based on taxanes (e.g., paclitaxel and docetaxel) remains the mainstay therapeutic strategy for both early stage and metastatic TNBC.^2^, ^3^, ^4^, ^5^ Unfortunately, despite an initial response, tumours inevitably develop resistance to taxane treatment. In the early stage, about 30% of patients with TNBC progress within five years, with a five‐year relapse‐free survival rate of about 70% and an overall survival rate of about 75%, which is much lower than that for ERBB2+ or HR+ breast cancer.^6^, ^7^ Therefore, determining the resistance mechanisms towards paclitaxel and attempting to overcome paclitaxel resistance is vitally important in clinical practice.

Paclitaxel exerts its anti‐tumour effect in a complex manner, including arresting microtubule assembly, overproduction of reactive oxygen species, and induction of an inflammatory response.^8^ In response to the various cellular stresses imposed by paclitaxel, cancer cells initiate apoptosis or gear cellular stress towards survival, acquiring paclitaxel resistance. Emerging evidence indicates that breast cancer cells develop multiple mechanisms to fight the stress induced by paclitaxel, such as increasing microtubule dynamics, adapting to increased oxidative stress, and upregulation of anti‐apoptotic proteins.^8^, ^9^, ^10^, ^11^, ^12^ Unfortunately, to date, no strategies have emerged to reverse paclitaxel resistance. Evidence suggests that mRNA processing and export are involved in inducing resistance to anti‐cancer strategies.^13^, ^14^, ^15^, ^16^, ^17^ However, the impact of mRNA export complexes on resistance to anti‐microtubule agents has rarely been investigated. Therefore, it is important to identify whether and which mRNA export subunit is associated with paclitaxel resistance in TNBC.

Aly/REF Export factor (ALYREF), a well‐characterized component of the transcription export (TREX) protein complex, participates in several steps of mRNA metabolism, including mRNA processing, mRNA stability, and mRNA nuclear–cytoplasmic transport.^18^, ^19^ ALYREF was significantly upregulated in several tumour tissues, including breast cancer.^20^ Recent reports showed that upregulated ALYREF correlated with tumour growth, disease progression, immune evasion, and poor survival.^20^, ^21^, ^22^, ^23^ However, whether ALYREF is involved in resistance to taxanes in TNBC, and its potential mechanisms, remains unknown. Biologically, ALYREF shuttles between the cytoplasm and the nucleus, and resides in the nuclear speckle.^13^, ^24^, ^25^ ALYREF is transported outside the nucleus during the mRNA export process.^26^, ^27^ The subsequent nuclear import of ALYREF is regulated by several factors. In addition to passive diffusion through the nuclear pore complex,^28^, ^29^ nuclear import of ALYREF is tightly regulated by the importin (IPO)‐mediated nuclear transport system.^30^, ^31^ However, several stresses were found to interfere with this import system.^32^ Therefore, it would be interesting to investigate whether and how paclitaxel treatment impairs ALYREF cytoplasmic‐nuclear shuttling, and the role of ALYREF in paclitaxel resistance.

Protein post‐translational modification (PTM) is one of the critical mechanisms that regulate protein subcellular distribution,^33^ and might play a role in regulating stress‐induced transport.^34^, ^35^ The E3 ligase ring finger protein 31 (RNF31), the core catalytic unit of the linear ubiquitin chain assembly complex (LUBAC),^36^ generates Met1‐linked linear ubiquitination of various protein substrates, such as nuclear factor kappa B (NF‐kB) essential modulator.^37^, ^38^ Previous studies suggested that elevated RNF31 expression accelerates tumour progression and treatment resistance.^39^, ^40^, ^41^ However, the cellular signalling substrates regulated by RNF31 and its biological function are incompletely understood.

Herein, we aimed to determine whether paclitaxel treatment impairs ALYREF cytoplasmic–nuclear shuttling, the mechanism, and the role of ALYREF and its RNF31‐mediated ubiquitination in paclitaxel resistance. We identified that in paclitaxel‐sensitive TNBC, ALYREF was trapped in the cytoplasm by paclitaxel treatment, while in paclitaxel‐resistant TNBC, ALYREF was efficiently transported into the nucleus to exert its function. This suggested that, in response to paclitaxel treatment, the proper localization of ALYREF induced paclitaxel resistance in TNBC. Mechanistically, in response to paclitaxel treatment, RNF31‐mediated ubiquitination of ALYREF efficiently facilitated ALYREF nuclear import via importin 13 (IPO13)‐mediated nuclear transport. This would enhance ALYREF‐mediated export of mRNAs encoding paclitaxel resistance‐related factors, including tubulin beta 3 class III (TUBB3), stathmin 1 (STMN1), and microtubule‐associated protein Tau (TAU), leading to paclitaxel resistance. Treatment with the RNF31 inhibitor Thiolutin overcame paclitaxel resistance and markedly impeded tumour progression in TNBC. These data revealed that RNF31‐mediated ubiquitination of ALYREF plays a vital role in TNBC paclitaxel resistance, highlighting RNF31 as a likely druggable target.

## 2 METHODS

### 2.1 Patient specimens

TNBC specimens were collected from patients who had undergone taxane‐based neoadjuvant or adjuvant chemotherapy. All patients were diagnosed at Sun Yat‐sen University Cancer Center. The study included 26 patients treated with neoadjuvant chemotherapy and 157 patients treated with adjuvant chemotherapy. Detailed clinical and pathological characteristics are provided in the Supporting Information Tables.

### 2.2 Immunohistochemistry

Immunohistochemical (IHC) staining was carried out on the TNBC tissue sections included in the study, utilizing anti‐ALYREF and anti‐RNF31 antibodies, as previously described.^33^ The integrated optical density (IOD) of the IHC staining was measured using Image‐Pro Plus Version 6.2 software (Media Cybernetics Inc.), with subgroup classification based on the median IOD value. Samples with IOD values equal to or exceeding the median were classified as the high‐expression group, while those with IOD values below the median were assigned to the low‐expression group. The full process of IHC staining is provided in the Supporting Information Materials.

### 2.3 Xenograft tumour models and treatment

Immune‐deficient nude mice were used to examine the in situ growth of MDA‐MB‐231 tumours. Mice were housed in barrier facilities on a 12 h light/dark cycle. The indicated cells (1 × 10^6^ cells/mouse) were injected into the mammary fat pads of nude mice (*n* = 6/group) to establish the orthotopic xenograft models. At 14 days after tumour induction, when the median tumour volumes reached approximately 200 mm^3^, dimethyl sulfoxide (DMSO), paclitaxel (PTX) (10 mg/kg), and Thiolutin (5 mg/kg) were intraperitoneally (i.p.) injected according to the indicated groups. Paclitaxel was added every 2 days for six times in total, and Thiolutin was added every day for five times in total. Tumour volumes were measured every 3 days and calculated using the following formula: tumour volume = 1/2 × length × width^2^. Once tumour‐bearing mice lost 10% of their weight, experienced cachexia, or the tumour volumes reached 1500 mm^3^, the mice were sacrificed, and the tumours were excised for further examination.

### 2.4 Patient‐derived organoid culture

Patient‐derived organoid cultures were established from histologically confirmed TNBC tissues obtained from biopsy specimens at Sun Yat‐sen University Cancer Center, with written consent provided by the patients before surgery. Tissue processing and organoid culture were performed as previously described.^42^ In brief, freshly resected tumour tissues were promptly processed upon receipt and cultured. Depending on the tumour size, the untrypsinized organoids were embedded in Matrigel. Once the Matrigel‐cell solution solidified in the 48‐well plates, the breast cancer organoid medium was added immediately and refreshed every 2 to 3 days, as appropriate. After 2 to 4 weeks of growth, TNBC organoids were harvested for quantitative real‐time reverse transcription PCR (qRT‐PCR) and western blotting. Trypsinization and passaging of organoids were carried out approximately every 14 to 21 days.

### 2.5 Drug response test of TNBC organoids

Organoids exhibiting high RNF31 expression and optimal conditions were selected for the drug sensitivity test. Of the five organoids analyzed, two (#1 and #2) were identified as having high RNF31 levels and were chosen for further drug response testing. PTX was administered at a concentration of 10 nM, while Thiolutin was applied at a concentration of 1 µM. Organoid images were captured using a Nikon Eclipse Ti2‐E microscope (Nikon). Cell viability was assessed using the CellTiter‐Glo 3D cell viability assay (Promega; G9683), following the manufacturer's protocol.

### 2.6 Statistical analysis

The SPSS software (version 25.0, IBM Corp.) was applied to perform all statistical analyses. Differential comparisons were carried out using Student's *t*‐test (two‐tailed), the Mann–Whitney *U* test (two‐tailed), the chi‐squared test, one‐way analysis of variance (ANOVA), or one‐way repeated measures ANOVA test, as appropriate. Survival comparisons were evaluated using the Kaplan–Meier survival method and a log‐rank test. Independent risk factors for survival were assessed using the Cox regression models. A *p‐*value of less than .05 was considered statistically significant.

## 3 RESULTS

### 3.1 ALYREF trafficking correlates with the response to PTX in triple‐negative breast cancer

**FIGURE 1:**
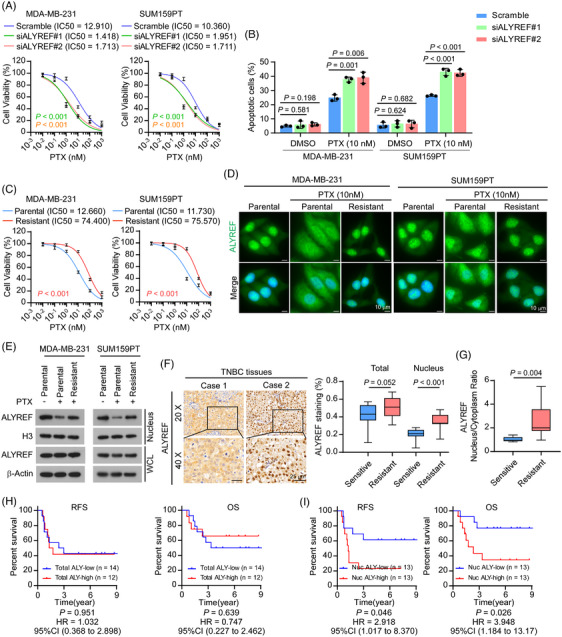
ALYREF trafficking correlates with the response to paclitaxel in triple‐negative breast cancer. (A) Cell viability in the indicated groups. (B) Flow cytometry‐mediated Annexin V assay showing the cell apoptosis rate in the indicated groups. (C) Cell viability in paclitaxel‐resistant and parental TNBC cells. (D) Representative images of immunofluorescence assays showing ALYREF subcellular distribution in the indicated groups. Paclitaxel (10 nM) or control was treated for 48 h before fixation. (E) Western blotting assay to detect ALYREF levels in nuclear extracts and the whole cell lysate in the indicated groups. (F) Representative images of ALYREF IHC staining of samples from patients with TNBC (left). Scale bar = 25 µm. Quantification of total and nuclear ALYREF staining from TNBC IHC compared between chemotherapy‐resistant (PD/SD, *n* = 15) and chemotherapy‐sensitive patients (CR/PR, *n* = 11) (right). (G) Comparison of the nuclear‐cytoplasm ratio of ALYREF in the indicated groups. (H) K–M plots showing the correlation of the total ALYREF staining score with relapse‐free survival and overall survival. (I) K–M plots showing the correlation of the nuclear ALYREF staining score with RFS and OS. IC50, half maximal inhibitory concentration. PTX, paclitaxel.

Previous studies reported that ALYREF shuttles between the nucleus and the cytoplasm.^19^, ^43^ However, the regulatory mechanisms of ALYREF function and its subcellular distribution in humans are not fully understood. To identify the importance and biological function of ALYREF in the treatment response of patients with TNBC, we first investigated the impact of ALYREF on different chemotherapy agents in TNBC cells. First, we established small interfering RNA (siRNA)‐mediated knockdown of *ALYREF* in TNBC cell lines, MDA‐MB‐231 and SUM159PT. The knockdown efficiency was validated using qRT‐PCR and western blotting (Figure S1A). Cell viability assays using cell counting kit‐8 (CCK8) were performed to evaluate cell viability in response to treatment with different chemotherapeutic drugs (epirubicin (EPX), cyclophosphamide (CTX) and PTX)^2^ in *ALYREF* knockdown cells. The results showed that *ALYREF* knockdown significantly reduced cell viability upon PTX treatment in MDA‐MB‐231 and SUM159PT cells (Figure 1A). However, the effect of *ALYREF* knockdown on EPX and CTX cytotoxicity was less significant, as the cell viability was only slightly decreased in ALYREF knockdown cells in comparison with that in the scramble siRNA control cells (Figure S1B). In addition, a flow cytometry‐mediated Annexin V assay showed that silencing of *ALYREF* significantly increased the apoptosis rate upon PTX treatment in both MDA‐MB‐231 and SUM159PT cells (Figure 1B; Figure S1C). To further confirm the role of ALYREF in PTX efficacy, we generated PTX‐resistant MDA‐MB‐231 and SUM159PT cell lines (MDA‐MB‐231‐R and SUM159PT‐R; Figure 1C) and detected ALYREF expression in PTX‐resistant and parental cells (MDA‐MB‐231‐P and SUM159PT‐P). Unexpectedly, both the mRNA and protein levels of ALYREF were comparable between PTX‐resistant and parental TNBC cells (Figure S1D), indicating that it was not the expression level of ALYREF, but possibly post‐translational processing, which might be involved in the PTX response in TNBC. We then detected ALYREF using an immunofluorescence assay in TNBC cells. As shown in Figure 1D and Figure S1E, in parental TNBC cells, ALYREF was primarily located in the nucleus, while in the presence of PTX, ALYREF was trapped in the cytoplasm. Importantly, in PTX‐resistant TNBC cells, ALYREF was mainly localized in the nucleus. Consistently, the distribution changes of ALYREF were validated by subcellular fraction extraction and subsequent western blotting: the nuclear‐localized ALYREF was reduced upon PTX treatment in parental cells but recovered to a level comparable to that in untreated cells in PTX‐resistant cells (Figure 1E). To validate the effect of ALYREF distribution on PTX resistance, we detected ALYREF distribution in 26 tumour tissues collected from patients with early‐stage TNBC treated with PTX‐based neoadjuvant chemotherapy. Tumour tissues were obtained after systemic treatment. Immunohistochemical (IHC) staining showed that the ALYREF level was slightly increased in chemotherapy‐resistant (progressive disease/stable disease, PD/SD) TNBC (*p = *.052; Figure 1F) compared with that in chemotherapy‐sensitive TNBC (complete response/partial response, CR/PR, according to the RECIST 1.1 standard).^44^ We observed that in the chemotherapy‐resistant group, ALYREF was mainly localized in the nucleus, while ALYREF was trapped in the cytoplasm with reduced nuclear levels in the chemotherapy‐sensitive group (Figure 1F). In addition, the nuclear‐to‐cytoplasm ratio of ALYREF was higher in the chemotherapy‐resistant group (Figure 1G). Noticeably, the correlation between the total ALYREF level and poorer relapse‐free survival (RFS) and overall survival (OS) was not statistically significant (*p* > .05; Figure 1H), while a high nuclear ALYREF level following systemic treatment correlated significantly with poorer RFS and OS in patients with TNBC (Figure 1I). Meanwhile, the nuclear ALYREF level did not correlate significantly with tumour stage or lymph node stage in TNBC (Figure S1F).

Taken together, these results indicated that ALYREF nuclear transport correlated substantially with a poor PTX response in TNBC.

### 3.2 RNF31‐mediated ALYREF ubiquitination facilitated ALYREF nuclear transport upon PTX treatment

**FIGURE 2:**
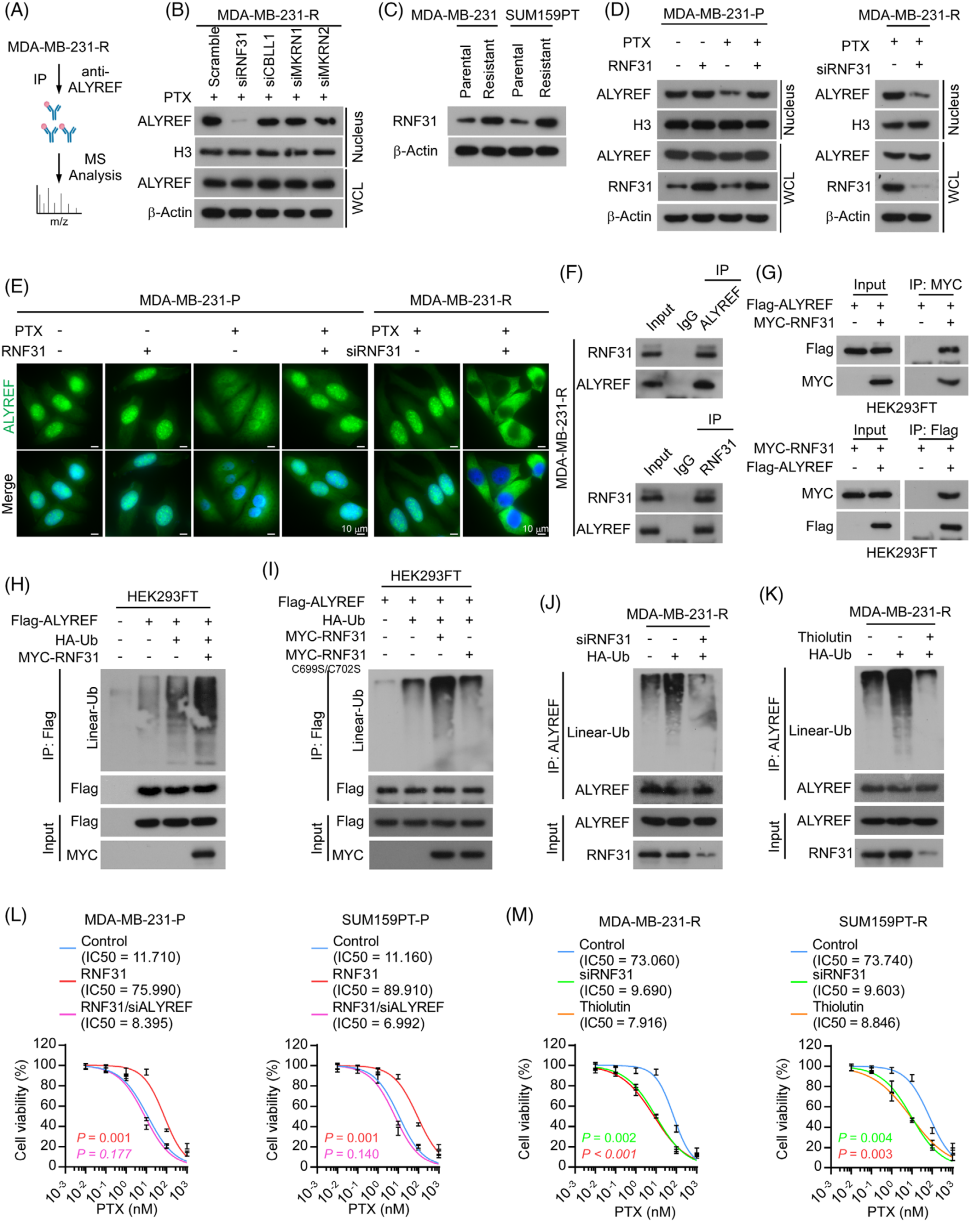
RNF31‐mediated ALYREF ubiquitination facilitated ALYREF nuclear transport upon paclitaxel treatment. (A) Diagram of immunoprecipitation/ mass spectrometry analysis (IP/MS) of the ALYREF interactome in paclitaxel‐resistant MDA‐MB‐231 cells using anti‐ALYREF antibodies. (B) Western blotting shows the nuclear level and total level of ALYREF upon siRNA‐mediated knockdown of *RNF31*, *CBLL1*, *MKRN1*, or *MKRN2* in MDA‐MB‐231‐R cells. (C) Western blotting showing the RNF31 level in paclitaxel‐resistant cells and parental TNBC cells. (D) The nuclear and total levels of ALYREF with indicated treatments in MDA‐MB‐231‐P cells, as detected through nuclear fraction extraction, followed by western blotting (left). Nuclear ALYREF level upon silencing *RNF31* in MDA‐MB‐231‐R cells, as detected using western blotting (right). (E) IF assay showing ALYREF subcellular distribution with the indicated treatments in MDA‐MB‐231 cells. Scale bar = 10 µm. (F) Endogenous reciprocal immunoprecipitation assays (Co‐IP) to determine the interaction between ALYREF and RNF31 in MDA‐MB‐231‐R cells. (G) Exogenous Co‐IP assays to determine the interaction between Flag‐tagged ALYREF and MYC‐tagged RNF31 in HEK293FT cells. (H) Flag‐tagged ALYREF, MYC‐tagged RNF31 and HA‐tagged Ub were expressed in HEK293FT cells, followed by IP assays with anti‐Flag beads to determine the linear ubiquitylation level of ALYREF. (I) Flag‐tagged ALYREF, MYC‐tagged RNF31 or active site mutant RNF31 and HA‐tagged Ub were expressed in HEK293FT cells, followed by IP assays with anti‐Flag beads to determine the linear ubiquitylation level of ALYREF. (J) A siRNA targeting *RNF31* was transfected in MDA‐MB‐231‐R cells, followed by IP assays with anti‐ALYREF beads to examine the linear ubiquitination of ALYREF. (K) Thiolutin (1 µM) was added to MDA‐MB‐231‐R cells, followed by IP assays using anti‐ALYREF beads to examine the linear ubiquitination of ALYREF. (L) Cell viability under the indicated treatments in MDA‐MB‐231‐P and SUM159PT‐P cells. (M) Cell viability under the indicated treatments in MDA‐MB‐231‐R and SUM159PT‐R cells.

Given the above findings for the subcellular distribution of ALYREF and its potential role in PTX resistance in TNBC, we next investigated the molecular mechanism through which ALYREF was transported from the cytoplasm to the nucleus under the stress conditions imposed by PTX. Currently, the nuclear transport pathway of ALYREF remains mostly unknown. To this end, we first performed immunoprecipitation followed by mass spectrometry analysis (IP/MS) to identify proteins that interact with ALYREF in PTX‐resistant‐TNBC cells (Figure 2A; Table S6).

Emerging evidence shows that protein post‐translational modification, specifically ubiquitination, plays a vital role in protein trafficking^34^, ^35^; therefore, we focused on the E3 ligases in the mass spectrometry assay, and found that several E3 ligases interacted with ALYREF, including RNF31, Cbl proto‐oncogene like 1 (CBLL1), Makorin ring finger protein 1 (MKRN1), and MKRN2. Assessment of protein levels in the nuclear fractions and whole cell lysates showed that siRNA‐mediated depletion of *RNF31*, but not *CBLL1*, *MKRN1*, or *MKRN2*, reduced the nuclear ALYREF level in PTX‐resistant TNBC cells (Figure 2B; Figure S2A). The effect of RNF31 on ALYREF nuclear trafficking and PTX resistance was then further examined.

The expression level of RNF31 was upregulated in PTX‐resistant cells compared with that in parental cells (Figure 2C). Exogenous overexpression of *RNF31* in parental TNBC cells showed that in the absence of PTX treatment, RNF31 scarcely influenced the nuclear localization of ALYREF. By contrast, upon PTX treatment, overexpression of *RNF31* increased the nuclear level of ALYREF in parental TNBC cells (Figure 2D; Figure S2B). Conversely, in PTX‐resistant cells, silencing *RNF31* dramatically reduced the nuclear ALYREF level under PTX treatment (Figure 2D, Figure S2B). An immunofluorescence assay showed that ectopic expression of *RNF31* relieved the cytoplasm‐trapped ALYREF and increased the nuclear ALYREF level in response to PTX treatment in parental TNBC cells, while knockdown of *RNF31* trapped ALYREF in the cytoplasm in PTX‐resistant TNBC cells (Figure 2E; Figure S2C,D). These data suggested that RNF31 promotes ALYREF nuclear translocation upon PTX administration.

Subsequently, we confirmed the interaction between ALYREF and RNF31 using endogenous and exogenous reciprocal immunoprecipitation. The results clearly showed that ALYREF interacted with RNF31 in PTX‐resistant TNBC cells (Figure 2F; Figure S2E) and HEK293FT cells (Figure 2G). RNF31 serves as the catalytic subunit of the E3 ubiquitin ligase complex LUBAC^36^; therefore, we detected the impact of RNF31 on ALYREF and observed that overexpressing *RNF31* enhanced the linear‐linked ubiquitination level of ALYREF (Figure 2H). Specifically, an active site mutant of RNF31 (C699S/C702S) failed to modulate the linear ubiquitination of ALYREF (Figure 2I). Then, we demonstrated that silencing *RNF31* (Figure 2J; Figure S2F) or blocking RNF31 activity using Thiolutin (an inhibitor of RNF31)^45^ (Figure 2K; Figure S2G) eliminated the linear ubiquitylation of ALYREF in PTX‐resistant TNBC cells. As expected, Thiolutin treatment reduced the nuclear ALYREF level dramatically in PTX‐resistant TNBC cells (Figure S2H). An in vitro assay showed that overexpressing *RNF31* substantially increased the cell viability and colony formation ability of parental TNBC cells under PTX treatment, while knockdown of *ALYREF* blocked the effect induced by *RNF31* overexpression in parental TNBC cells (Figure 2L; Figure S2I). Silencing *RNF31* or Thiolutin treatment both inhibited the cell viability and colony formation ability of PTX‐resistant TNBC cells upon PTX treatment (Figure 2M; Figure S2J).

The above results showed that RNF31 ubiquitinated ALYREF and facilitated ALYREF nuclear import upon PTX treatment, consequently mediating PTX resistance in TNBC cells.

### 3.3 RNF31 enhanced ALYREF nuclear transport via importin 13

**FIGURE 3:**
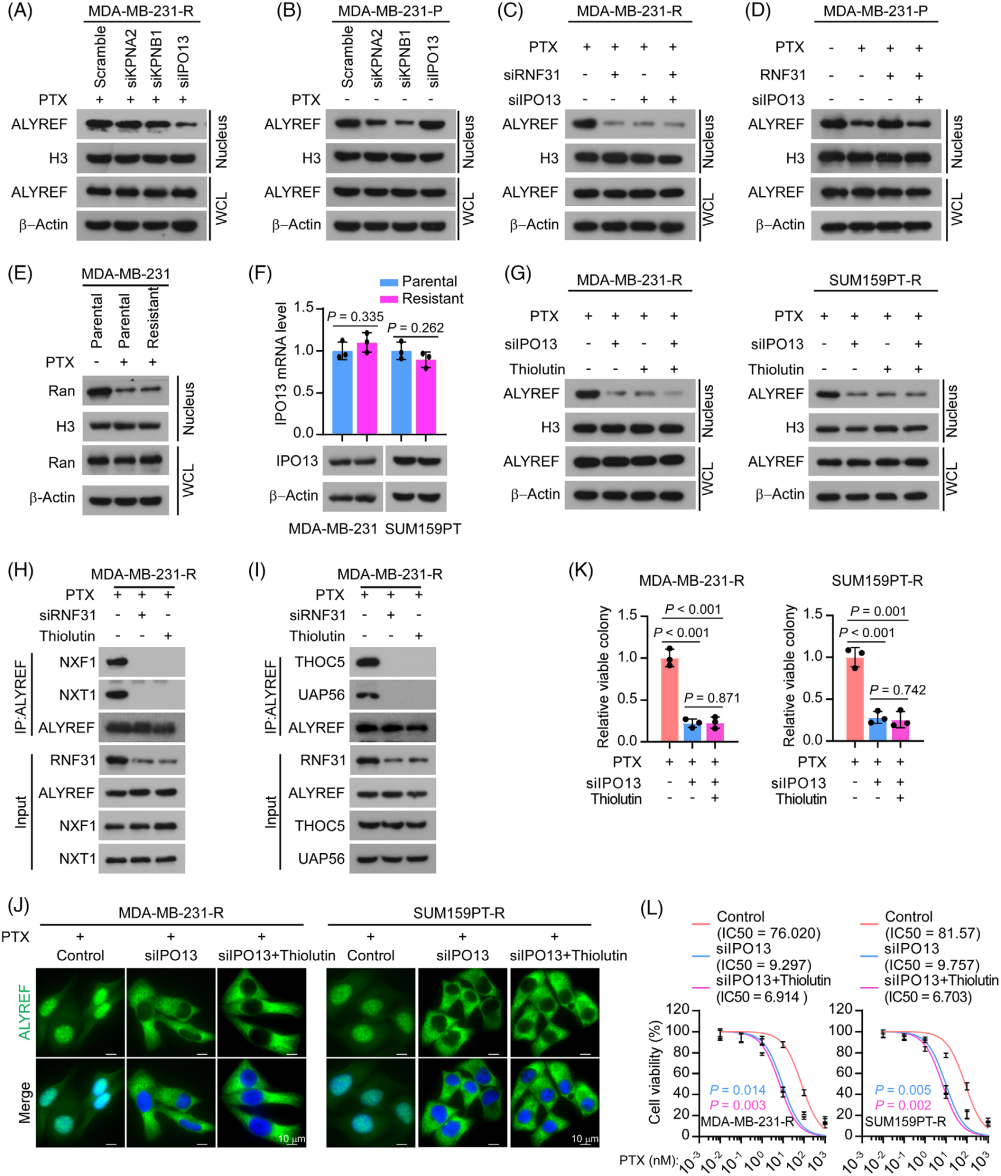
RNF31 enhanced ALYREF nuclear transport via importin 13. (A) Western blotting showing the nuclear and total levels of ALYREF upon siRNA‐mediated knockdown of *IPO13*, *KPNA2*, or *KPNB1* in MDA‐MB‐231‐R cells. (B) Western blotting showing the nuclear and total level of ALYREF upon siRNA‐mediated knockdown of *IPO13*, *KPNA2*, or *KPNB1* in MDA‐MB‐231‐P cells without paclitaxel treatment. (C) siRNA‐mediated knockdown of *RNF31* or *IPO13* decreased the nuclear ALYREF level in MDA‐MB‐231‐R cells. (D) Nuclear ALYREF levels in the indicated groups, as detected using western blotting. Cells were treated with paclitaxel (10 nM) for 48 h before harvest. (E) The nuclear Ran level in the indicated groups detected using western blotting. (F) The mRNA and protein levels of IPO13 as detected in parental and paclitaxel‐resistant MDA‐MB‐231 and SUM159PT cells. (G) Total and nuclear ALYREF levels under the indicated treatments, as detected in paclitaxel‐resistant MDA‐MB‐231 and SUM159PT cells. (H) IP‐western blotting determination of the interaction between ALYREF and NXF1 or NXT1 in MDA‐MB‐231‐R cells upon *RNF31* knockdown or Thiolutin (1 µM) treatment. (I) IP‐western blotting determination of the interaction between ALYREF and THOC5 or UAP56 in MDA‐MB‐231‐R cells with the indicated treatments. (J) Representative IF images showing the ALYREF subcellular distribution under the indicated treatments in MDA‐MB‐231‐R cells and SUM159PT‐R cells. Scale bar = 10 µm. (K) Colony formation assay showing the surviving colony numbers of MDA‐MB‐231‐R and SUM159PT‐R cells under the indicated treatments. (L) Cell viability of MDA‐MB‐231‐R and SUM159PT‐R cells under the indicated treatments.

We then investigated the pathway by which RNF31 mediates ALYREF nuclear translocation upon PTX treatment. Nuclear import of proteins is predominantly carried out by the IPO superfamily, which relies on the small GTPase Ran.^46^, ^47^ The collapse of the RanGTP‐RanGDP concentration gradient across the nuclear membrane dramatically blocks Ran‐dependent IPO shuttling across the nuclear membrane.^46^, ^47^, ^48^, ^49^, ^50^, ^51^ Emerging evidence demonstrates that various types of cellular stress, including heat stress and oxidative stress, destroy the RanGTP‐RanGDP gradient, which inactivates the IPO system and traps proteins in the cytoplasm. Karyopherin subunit Beta 1 (KPNB1 or importin β1) and KPNA2 (importin α1) are well‐investigated karyopherins that transport a variety of cargoes in a Ran‐dependent manner.^52^ Interestingly, our anti‐ALYREF IP/MS assay showed that KPNB1 and KPNA2 interacted with ALYREF in PTX‐resistant cells (Table S6). Besides, IPO13, a bidirectional nuclear transporter, was found recently to function efficiently under several stress conditions in a Ran‐independent manner.^50^, ^51^ Therefore, we investigated the function of different karyopherins in ALYREF trafficking under PTX‐induced stress. siRNA knockdown of *IPO13* dramatically reduced the nuclear ALYREF level in PTX‐resistant TNBC cells, while silencing *KPNB1* or *KPNA2* only slightly altered the nuclear ALYREF level in PTX‐resistant TNBC cells (Figure 3A; Figure S3A). Notably, in parental TNBC cells without PTX treatment, silencing *KPNB1* or *KPNA2* significantly reduced the nuclear ALYREF level, while the knockdown of *IPO13* barely influenced the nuclear ALYREF level (Figure 3B; Figure S3B).

In PTX‐resistant cells, the knockdown of either *RNF31* or *IPO13* decreased the nuclear ALYREF level (Figure 3C). In parental TNBC cells, ectopic expression of *RNF31* rescued the decreased nuclear ALYREF level induced by PTX treatment, while silencing *IPO13* eliminated the effect of ectopic RNF31 expression (Figure 3D). Subsequently, we confirmed the interaction between ALYREF and IPO13 exogenously: reciprocal immunoprecipitation‐western blotting showed that ectopic expression of RNF31 triggered the interaction between ALYREF and IPO13, while the absence of RNF31 attenuated the interaction between ALYREF and IPO13 in HEK293FT cells (Figure S3C). RanGTP is mainly localized in the nucleus, whereas RanGDP is predominantly located in the cytoplasm.^46^, ^47^ The collapse of the RanGTP‐RanGDP concentration gradient by stress reduced the RanGTP level and trapped Ran in the cytoplasm.^49^, ^51^ As expected, upon PTX treatment, the nuclear Ran level was dramatically reduced (Figure 3E), and the RanGTP level was dramatically decreased. RAN binding protein 1 (RanBP1) was found to bind to RanGTP but not RanGDP^53^, ^54^ (Figure S3D). Notably, both the mRNA and protein levels of IPO13 were comparable between PTX‐resistant and parental TNBC cells (Figure 3F). The above results indicated that in the absence of PTX treatment, KPNB1 and KPNA2 function as the predominant transporters of ALYREF in TNBC, while upon PTX stress, IPO13, but not KPNB1 or KPNA2, serves to efficiently transport ALYREF into the nucleus in TNBC cells.

Individual inhibition of either IPO13 or RNF31 dramatically reduced the nuclear ALYREF level in PTX‐resistant TNBC cells, while dual inhibition did not produce an enhanced effect on the nuclear ALYREF level, indicating that RNF31 and IPO13 regulate the nuclear ALYREF level in the same pathway (Figure 3G). We also noticed that the interaction of ALYREF with mRNA export complex components (NXF1, NXT1, THOC5, and UAP56)^43^, ^55^, ^56^ was reduced by silencing *RNF31* or adding Thiolutin in PTX‐resistant TNBC cells (Figure 3H,I). Besides, silencing *IPO13*, and *IPO13* silencing in combination with Thiolutin, reduced the nuclear ALYREF level in PTX‐resistant TNBC cells (Figure 3J; Figure S3E). However, combining Thiolutin with *IPO13* silencing did not show an additive effect on reducing the nuclear ALYREF level, further demonstrating that RNF31 and IPO13 function in the same pathway to regulate ALYREF nuclear translocation upon PTX treatment (Figure 3J; Figure S3E). Consistently, silencing *IPO13*, or *IPO13* silencing combined with Thiolutin, dramatically inhibited cell viability and colony formation ability upon PTX treatment in PTX‐resistant TNBC cells, while there was no significant difference between the effects silencing *IPO13* and the combination of silencing *IPO13* and Thiolutin (Figure 3K,L; Figure S3F).

Taken together, the above data indicated that RNF31 facilitated ALYREF nuclear transport in response to PTX treatment in an IPO13‐dependent manner.

### 3.4 RNF31 facilitated ALYREF‐mediated PTX resistance in TNBC in vitro

**FIGURE 4:**
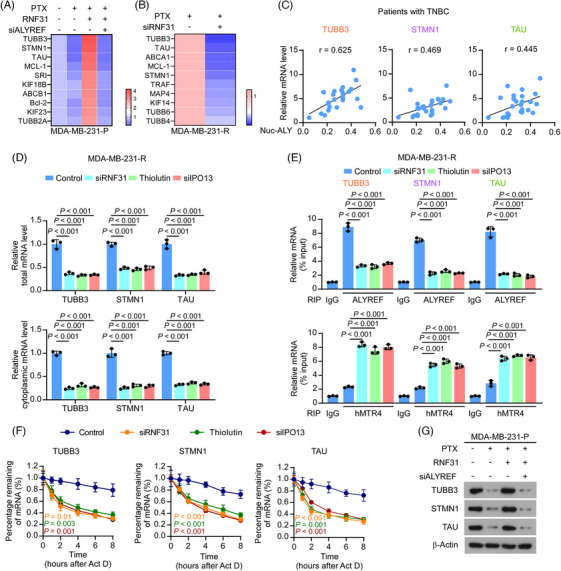
RNF31 facilitated ALYREF‐mediated paclitaxel resistance in TNBC in vitro. (A) Heatmap of top 10 factors related to paclitaxel resistance with the most significant fold changes upon RNF31 and ALYREF expression in MDA‐MB‐231‐P cells. (B) Heatmap of top 10 factors related to paclitaxel resistance with the most significant fold changes upon silencing of *RNF31* in MDA‐MB‐231‐R cells. (C) Pearson correlation assay showing the correlation between nuclear ALYREF scoring and TUBB3, STMN1, and TAU expression in 26 samples from patients with TNBC who received taxane‐based neoadjuvant chemotherapy. (D) Total and cytoplasmic mRNA levels of *TUBB3*, *STMN1*, and *TAU* in cells under the indicated treatments, as detected using qRT‐PCR. (E) RIP‐PCR using anti‐ALYREF and anti‐hMTR4 antibodies showing the mRNA binding ability between ALYREF or hMTR4 and *TUBB3*, *STMN1*, and *TAU* in the indicated groups. (F) The half‐life of *TUBB3*, *STMN1*, and *TAU* mRNA, as detected using qRT‐PCR at set time points after ActD treatment in the indicated groups. ActD, actinomycin. (G) Protein levels of TUBB3, STMN1, and TAU detected using western blotting in the indicated groups.

To investigate whether RNF31 is involved in ALYREF‐mediated PTX‐resistance in TNBC, we first examined the mRNA abundance of PTX‐resistance‐related factors upon PTX treatment^8^ using qRT‐PCR in PTX‐resistant and parental MDA‐MB‐231 cells. In parental cells, PTX treatment dramatically reduced the mRNA levels of multiple factors related to PTX resistance. Overexpressing *RNF31* rescued the reduced level of PTX‐resistance‐related factors in parental cells, specifically upregulating the expression level of microtubule protein TUBB3, microtubule‐binding protein STMN1, and TAU. Blocking ALYREF expression eliminated the effect of ectopic *RNF31* expression on parental cells upon PTX treatment (Figure 4A). In addition, in PTX‐resistant cells, silencing of *RNF31* decreased the expression of PTX‐resistance‐related factors (Figure 4B). Three PTX‐resistance‐related factors, TUBB3, STMN1, and TAU, were selected for further exploration because their expression changes were most significant in response to ectopic *RNF31* expression.

Compared with those in parental TNBC cells, TUBB3, STMN1, and TAU levels were substantially increased in PTX‐resistant TNBC cells (Figure S4A,B). Consistently, upregulated TUBB3, STMN1, and TAU levels were observed in patients with TNBC who were resistant to PTX‐based chemotherapy (Figure S4C). The expression levels of TUBB3, STMN1, and TAU correlated significantly with nuclear ALYREF levels in patients with TNBC (Figure 4C). Furthermore, blocking *RNF31* with an siRNA or Thiolutin, or silencing *IPO13*, decreased both the total and cytoplasmic mRNA levels of *TUBB3*, *STMN1*, and *TAU*, as validated by nuclear‐cytoplasmic fractionation and subsequent qRT‐PCR (Figure 4D). ALYREF acts as an mRNA binding and mRNA export adaptor; therefore, we next explored the effect of RNF31 and IPO13 on ALYREF binding to mRNA. Anti‐ALYREF antibody‐mediated RNA immunoprecipitation (RIP)‐PCR showed that blocking RNF31 and silencing *IPO13* significantly decreased the level of ALYREF binding to *TUBB3*, *STMN1*, and *TAU* mRNAs in PTX‐resistant MDA‐MB‐231 cells (Figure 4E). By contrast, blocking RNF31 and silencing *IPO13* promoted the binding of *TUBB3*, *STMN1*, and *TAU* mRNA by Mtr4 exosome RNA helicase (MTREX, also known as hMTR4), a key marker of the mRNA degradation complex within the nucleus^57^ (Figure 4E). In addition, blocking RNF31 and silencing *IPO13* dramatically shortened the half‐life of *TUBB3*, *STMN1*, and *TAU* mRNA in PTX‐resistant MDA‐MB‐231 cells (Figure 4F). Besides, in parental cells, PTX treatment dramatically reduced the protein levels of TUBB3, STMN1, and TAU, and overexpressing *RNF31* rescued the reduced levels of TUBB3, STMN1, and TAU in parental cells (Figure 4G). Meanwhile, blocking *ALYREF* expression eliminated the effect of ectopic *RNF31* expression on parental cells upon PTX treatment (Figure 4G). The above results showed that RNF31 maintained ALYREF's mRNA binding and exporting functions; therefore, we further examined whether ALYREF affected RNF31 expression in response to PTX treatment. As shown in Figure S4D, in PTX‐resistant cells, silencing *ALYREF* and *IPO13* decreased RNF31 mRNA and protein levels, indicating that RNF31 and ALYREF form a positive feedback loop in PTX‐resistant TNBC cells.

Taken together, the above results demonstrated that RNF31 facilitated ALYREF‐induced PTX resistance by upregulating the expression of PTX‐resistance‐related factors.

### 3.5 RNF31 deficiency reversed PTX resistance in TNBC in vivo

**FIGURE 5:**
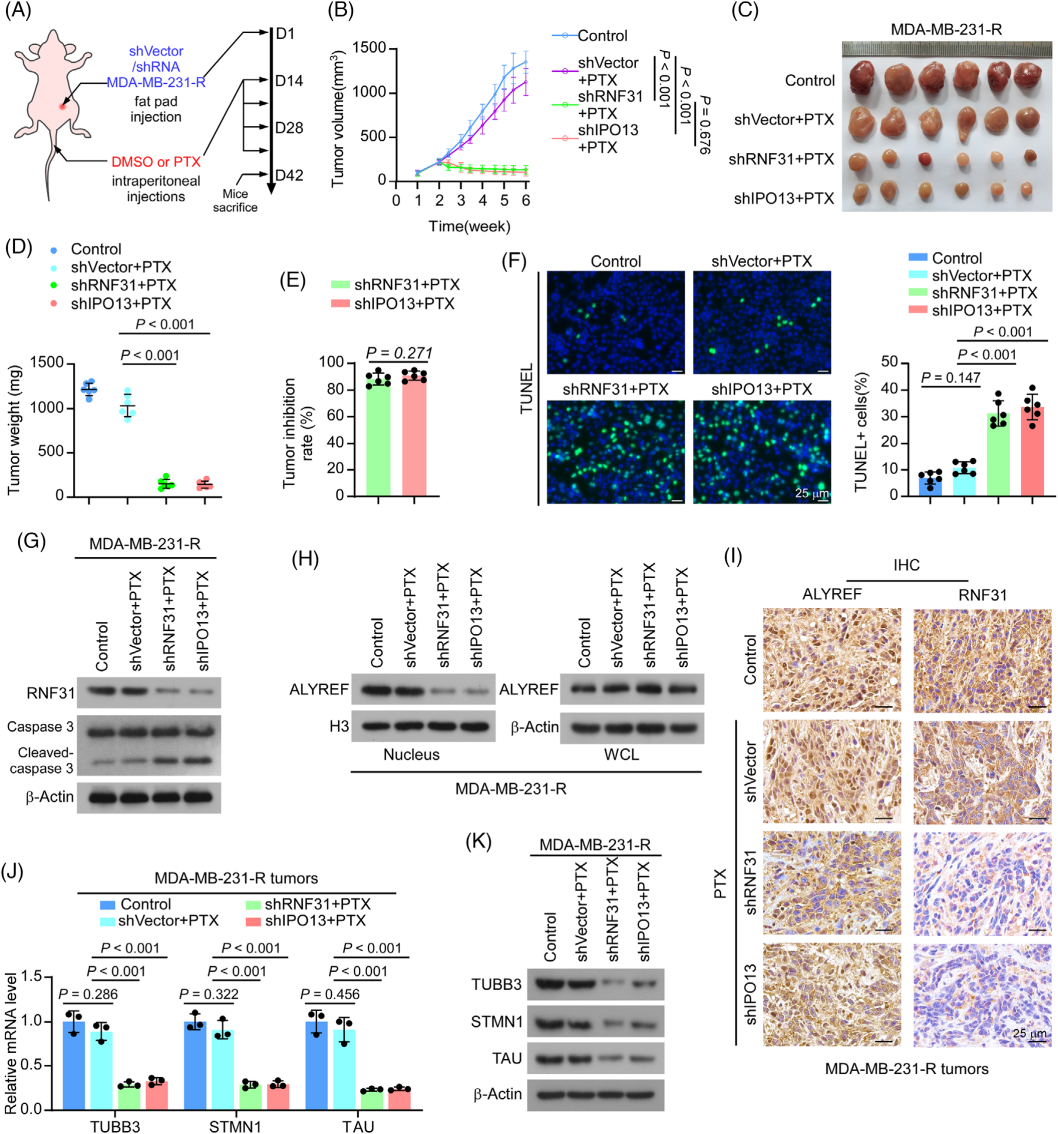
RNF31 deficiency reversed paclitaxel resistance in TNBC in vivo. (A) Schedule of tumour cell inoculation and PTX treatment in the nude mouse model. (B) Tumour volume curves for the indicated groups. (C) Images of tumours from the indicated groups. (D) Tumour weight (mg) in the indicated groups. (E) Tumour inhibition rate of the indicated groups relative to that of the shVector+PTX group. The formula (VshRNF31/shIPO13+PTX − VshVector+PTX)/ VshVector+PTX was applied to calculate the tumour inhibition rate. (F) Representative images of TUNEL staining and the quantification of apoptotic positive cells from the indicated groups. Scale bar = 25 µm. (G) Cleaved caspase 3 protein levels in the indicated groups, as detected using western blotting. (H) Total and nuclear ALYREF levels detected using western blotting in the indicated groups. (I) Representative IHC images of ALYREF and RNF31 in the indicated groups. Scale bar = 25 µm. (J) mRNA levels of *TUBB3*, *STMN1*, and *TAU*, as detected using qRT‐PCR in the indicated groups. (K) Protein levels of TUBB3, STMN1, and TAU detected using western blotting in the indicated groups.

To further determine whether RNF31 is involved in PTX resistance in TNBC, we examined the effect of RNF31 on the PTX response in a TNBC xenograft model. To this end, *RNF31* knockdown and *IPO13* knockdown PTX‐resistant MDA‐MB‐231 cells were injected into the breast fat pad of nude mice for a tumour growth assay (Figure 5A). Notably, deficiency of RNF31 and IPO13 greatly impaired tumour progression upon PTX administration, as indicated by significantly reduced increases in the size and weight of the xenograft tumours (Figure 5B–E). The tumours in the *IPO13* knockdown plus paclitaxel group were slightly smaller than those in the *RNF31* knockdown plus paclitaxel group, while the difference was not statistically significant (*p *= .676) (Figure 5B). These observations were further confirmed by a terminal deoxynucleotidyl transferase nick‐end‐labelling (TUNEL) assay and cleaved caspase‐3 detection, which showed much higher levels of TUNEL staining and cleaved caspase 3 levels in tumour tissues of the *RNF31* knockdown and *IPO13* knockdown groups, compared with those in the shVector plus PTX group (Figure 5F,G). The remaining tumours also showed strongly reduced nuclear ALYREF levels in tumour tissues of the *RNF31* knockdown and *IPO13* knockdown groups, as detected by western blotting and IHC assays (Figure 5H,I). Consistently, the mRNA and protein levels of TUBB3, STMN1, and TAU were dramatically reduced in the tumour tissues of the *RNF31* knockdown and *IPO13* knockdown groups treated with PTX (Figure 5J,K).

This in vivo model provided evidence that RNF31 deficiency significantly overcame PTX resistance in TNBC.

### 3.6 Inhibition of RNF31 activity increased PTX sensitivity in TNBC

**FIGURE 6:**
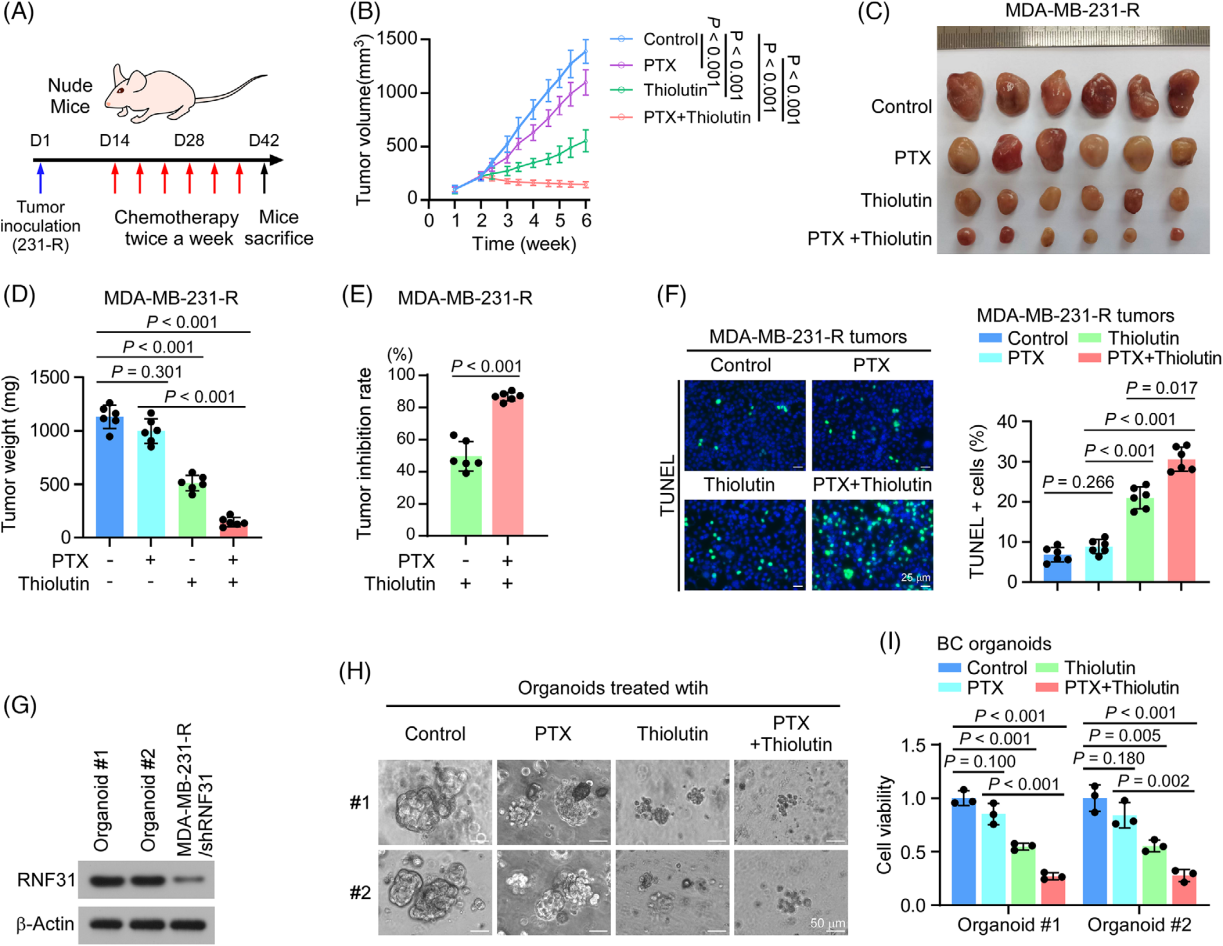
Inhibition of RNF31 activity increased paclitaxel sensitivity in TNBC. (A) Schedule of tumour cell inoculation and treatment strategies in the nude mouse model. (B) Tumour volume curves for the indicated groups. (C) Images of tumours from the indicated groups. (D) Tumour weight (mg) of the indicated groups. (E) Tumour inhibition rate of the indicated groups relative to that of the control group. The tumour inhibition rate of the indicated groups is relative to that of the PTX group. The formula (VThiolutin ± PTX − VPTX)/VPTX was applied to calculate the tumour inhibition rate. (F) Representative images of TUNEL staining and the quantification of apoptotic positive cells from the indicated groups. Scale bar = 25 µm. (G) Western blotting detection of the RNF31 level in two patient‐derived organoids, and RNF31‐silenced MDA‐MB‐231‐R cells. (H) Representative images of two patient‐derived organoids with the indicated treatments. Scale bar = 50 µm. (I) Cell viability in two patient‐derived organoids under the indicated treatments, as detected using the Glo 3D assay.

We further sought to translate these observations in vivo and in patient‐derived organoids. PTX‐resistant MDA‐MB‐231 cells were injected into the breast fat pad of nude mice to induce tumour formation. Then the mice were separated randomly into four groups: control, PTX treatment, Thiolutin treatment, and PTX plus Thiolutin treatment, respectively (Figure 6A). Notably, PTX plus Thiolutin dramatically inhibited tumour growth, as shown by the smaller tumour size and delayed tumour growth, while Thiolutin monotherapy also showed some tumour inhibition (Figure 6B–E). These observations were confirmed by a TUNEL assay for cell death, which showed a much higher TUNEL signal in tumour group treated with PTX plus Thiolutin than in the other groups (Figure 6F).

To further determine the synergistic effect of Thiolutin and PTX in TNBC, we treated TNBC patient‐derived organoids with PTX and Thiolutin. Two TNBC‐derived organoids expressing a high level of RNF31 were selected for drug evaluation (Figure 6G; Figure S5A). Compared with PTX single agent treatment, administration of PTX plus Thiolutin dramatically inhibited tumour growth in both organoids, while Thiolutin monotherapy slightly reduced organoid tumour growth (Figure 6H,I).

These results indicated that Thiolutin enhanced the sensitivity of TNBC to PTX therapy.

### 3.7 Clinical relevance of RNF31 in patients with TNBC

**FIGURE 7:**
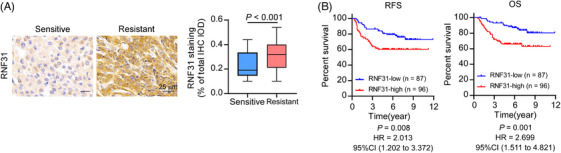
Clinical relevance of RNF31 in patients with TNBC. (A) Representative IHC images of RNF31 staining in samples from 183 patients with TNBC who received paclitaxel‐based chemotherapy (left). Statistical analysis of the RNF31 score in the 183 TNBC samples. Scale bar = 25 µm. (B) Kaplan–Meier survival curves for RFS and OS compared in the RNF31 high and RNF31 low groups. RFS, relapse‐free survival; OS, overall survival.

Finally, we assessed RNF31 expression in patients with TNBC. Immunohistochemical staining was performed on 183 patients with TNBC who received PTX‐based neoadjuvant (*n* = 26) or adjuvant (*n* = 157) chemotherapy. The IHC staining and correlation analysis revealed that RNF31 expression was higher in patients with TNBC who were resistant (relapse‐free survival < 5 years) to PTX‐based chemotherapy than in patients with TNBC who were sensitive (relapse‐free survival ≥5 years) to PTX‐based chemotherapy (Figure 7A). Patients with TNBC with high RNF31 levels who received PTX‐based neoadjuvant or adjuvant chemotherapy experienced poorer RFS and OS (Figure 7B). The clinical characteristics of the 183 patients included in the survival analysis are listed in Tables S1–S3. Univariate and multivariate analyses of Cox regression models showed that the RNF31 level was an independent predictive factor for OS for patients with TNBC who received PTX‐based neoadjuvant or adjuvant chemotherapy (hazard ratio [HR] = 2.790; 95% confidence interval [CI] 1.467–5.305; Tables S4 and S5).

## 4 DISCUSSION

Our study demonstrated that RNF31 facilitated ALYREF‐mediated PTX resistance in TNBC. Mechanistically, RNF31 efficiently promoted ALYREF nuclear transport via IPO13 in response to PTX treatment, subsequently enhancing the export of mRNAs encoding PTX‐resistance‐related factors, including TUBB3, STMN1, and TAU. By contrast, inhibiting RNF31 trapped ALYREF in the cytoplasm and induced TNBC cell death upon PTX treatment. The RNF31 inhibitor Thiolutin, which significantly blocked RNF31 activity, reversed PTX resistance in TNBC. Thus, our study provided a pharmacological strategy to overcome PTX resistance in patients with TNBC.

PTX‐based chemotherapy has been the backbone of TNBC treatment for decades; however, becoming refractory to PTX‐based systemic therapy remains a major obstacle in TNBC treatment.^2^ Thus, overcoming PTX resistance remains an unsolved problem. Herein, we showed that ALYREF, an mRNA export adaptor, plays a pivotal role in PTX resistance in TNBC. Accumulating evidence indicates that ALYREF expression correlates markedly with tumour growth and drug resistance. Overexpression of ALYREF was found to promote cancer progression by mediating RNA m5C modification in hepatocellular carcinoma.^21^ High expression of ALYREF was found to selectively regulate the short isoform of nuclear paraspeckle assembly transcript 1 (NEAT1) to impact energy metabolism and promote tumour growth in breast cancer.^20^ Moreover, in ovarian cancer, ectopic ALYREF expression conferred resistance to DNA‐damaging therapeutic agents such as platinum or poly (ADP‐ribose) polymerase (PARP) inhibitors, indicating that ALYREF is a pivotal factor in regulating anti‐cancer responses.^17^ Recently, ectopic expression of ALYREF was found to induce immune evasion in pancreatic ductal adenocarcinoma.^23^ Interestingly, our study found that ALYREF was closely related to PTX resistance in TNBC. Silencing *ALYREF* dramatically increased PTX sensitivity, while ALYREF mRNA and protein levels were not statistically different between PTX‐resistant and parental TNBC cells, indicating that the post‐translational modification and the subcellular distribution of ALYREF might participate in the response to PTX. ALYREF shuttles between the nucleus and cytoplasm; therefore, orchestration of ALYREF transport is critical in exerting its function. Yang et al.^26^ showed that NOP2/Sun RNA methyltransferase 2 (NSUN2) modulates ALYREF's shuttling between the nucleus and the cytoplasm, such that *NSUN2* knockdown increased ALYREF nuclear speckle retention, decreased the ALYREF cytoplasmic level, and reduced its ability to export its cargoes.^26^ By contrast, our study demonstrated that efficient transport of ALYREF into the nucleus, instead of trapping it in the cytoplasm upon PTX treatment, could retain the function of ALYREF in response to stress. This resulted in increased nuclear export and expression of several PTX‐resistance‐related factors, such as TUBB3, STMN1, and TAU, and induced PTX resistance. Noticeably, blocking ALYREF nuclear transport dramatically increased PTX sensitivity in TNBC, both in vivo and in patient‐derived organoids.

However, we know little about the mechanisms of ALYREF nuclear transport under stress conditions. The nuclear shuttling of ALYREF is carried out by the IPO superfamily. Zenklusen et al.^30^ reported that Yra1, the homolog of ALYREF in yeast, was transported to the nucleus by the IPObeta family members Pse1p and Kap123p. Herein, we found that in the absence of PTX treatment, ALYREF nuclear transport is mediated by importins KPNA2 and KPNB1, but not IPO13. By contrast, in PTX‐resistant cells treated with PTX, IPO13 functions in the nuclear transport of ALYREF, with little input from KPNB1 or KPNA2. The transport function of the IPO family relies on the small GTPase Ran.^46^, ^47^ The collapse of the RanGTP‐RanGDP concentration gradient across the nuclear envelope resulted in a dramatic mislocalization of the IPO proteins and blocked the IPO‐mediated nuclear transport of cargo proteins.^48^ Research showed that multiple environmental stresses could disrupt the RanGTP gradient, including oxidative stress and heat shock, causing malfunction of the Ran‐dependent IPO transport system.^32^ Herein, we observed that PTX treatment induced the collapse of the RanGTP‐RanGDP concentration gradient and disabled the function of KPNB1 and KPNA2 in TNBC, indicating that in PTX‐resistant TNBC cells, there is an alternative pathway to efficiently transport ALYREF under the stress conditions induced by PTX. Our results revealed that in PTX‐resistant TNBC, IPO13 efficiently drives the nuclear transport of ALYREF in cells under PTX‐induced stress. As a unique member of the IPO beta superfamily, IPO13's function is not absolutely dependent on the RanGTP gradient.^50^, ^51^ Unlike other IPObeta proteins, whose functions were impaired under stress conditions, but recovered inefficiently without stress, IPO13 is able to transport its cargoes efficiently under various stress conditions.^50^, ^51^ Notably, we did not observe a significant alteration in the expression level of IPO13 in parental and PTX‐resistant cells; therefore, it is probable that alternative mechanisms participate in regulating IPO13‐mediated nuclear import under these conditions.

Protein‐protein interactions and protein subcellular localization rely on PTM, suggesting that PTM is involved in IPO13‐mediated ALYREF nuclear transport in response to PTX treatment.^34^, ^35^, ^58^, ^59^, ^60^ RNF31 is the catalytic subunit of the LUBAC.^36^ It has been reported that RNF31 plays a multifaceted role in tumour proliferation, cell death, and the development of chemotherapy resistance in several malignancies. For example, RNF31 activated pro‐survival NF‐kB signalling and limited the activation of receptor‐interacting serine/threonine kinase 1 (RIPK1) via the linear ubiquitination of NF‐kB essential modifier (NEMO) and RIPK1, which further suppressed tumour necrosis factor (TNF)‐induced cell death.^39^ In addition, a study found that enforced expression of RNF31 protected cancer cells from DNA‐damage‐induced apoptosis, leading to resistance to cisplatin.^61^ In ER‐positive breast cancer, RNF31 could activate estrogen signalling via monoubiquitination to facilitate tumour growth.^62^ Meanwhile, in TNBC, RNF31 was found to exert a tumour suppressive role via K48 linkage of the Yes‐associated protein (YAP) for degradation, which reduces Hippo signalling target gene expression.^41^ These results indicated that RNF31 functions in various ways in different settings. Notably, the protein substrates of RNF31 and their biological significance are poorly understood.^45^ Our study further revealed an intriguing function of RNF31 as a potent factor associated with PTX resistance in TNBC. We identified ALYREF as a bona fide substrate of RNF31. RNF31 binds to and linearly ubiquitinates ALYREF, which facilitates ALYREF nuclear transport via IPO13, leading to enhanced expression of PTX‐resistance‐related factors. The conformational changes of ALRREF induced by RNF31‐mediated ubiquitination and the underlying mechanism of ALYREF's interaction with IPO13 warrant further investigation.

We then evaluated the therapeutic potential of blocking the RNF31/ALYREF interaction to reverse PTX refractory in TNBC. Given that ALYREF has a pivotal role in both pathological and biological processes, blocking ALYREF would overcome PTX resistance, but with inevitable severe side effects. Therefore, we focused on blocking RNF31 in an attempt to enhance the sensitivity of TNBC to PTX. Several inhibitors, such as Gliotoxin,^63^ Thiolutin,^64^, ^65^ and HOIPINs,^40^, ^66^ were found to inhibit RNF31 activity. In this study, we evaluated the potential to translate the above findings to preclinical in vivo and patient‐derived organoid settings. Our data clearly demonstrated that Thiolutin dramatically diminished the ALYREF nuclear level and reversed PTX resistance in TNBC, suggesting that inhibition of RNF31 activity might be a valid therapeutic target for TNBC. To further assess the synergistic effect of Thiolutin and PTX on TNBC treatment, we treated patient‐derived organoids with Thiolutin combined with PTX. For TNBC with a high level of RNF31, Thiolutin plus PTX substantially reduced tumour growth. However, these results should be interpreted with caution, because Thiolutin is not a specific inhibitor of RNF31. Thiolutin showed potent anti‐angiogenesis activity and induced cell apoptosis.^64^ Indeed, the off‐target effect of Thiolutin is also a promising target to inhibit tumours; however, further study is warranted to determine the anti‐cancer potential of RNF31 inhibition.

In conclusion, our study demonstrated that cytoplasmic–nuclear shuttling of ALYREF is associated with the response to PTX in TNBC. High levels of RNF31 indicated a poor response to PTX treatment and a dismal prognosis by facilitating ALYREF nuclear transport. However, inhibiting RNF31 activity re‐sensitized PTX‐resistant TNBC to PTX treatment. In a future study, we will investigate the clinical potential of RNF31 inhibition in preclinical and clinical settings.

## AUTHOR CONTRIBUTIONS

Shumei Huang, Dongni Shi, Shuqin Dai, and Xingyu Jiang performed the majority of the experimental work including data collection and analysis. Muwen Yang and Boyu Chen conducted the qRT‐PCR, western blotting, and immunofluorescence staining. Lingzhi Kong, Xuwei Chen, Rui Wang, Lixin He, and Pinwei Deng were responsible for tissue collection, patient information gathering, immunohistochemistry staining, and survival analysis. Xiangfu Chen conducted the immunoblotting analysis, plasmid constructions, and immunoprecipitation assays. Shumei Huang, Xiangfu Chen, Chuyong Lin, and Shuqin Dai conducted animal studies. Shumei Huang and Dongni Shi were responsible for cell culture, while Shuqin Dai and Xingyu Jiang carried out the in vitro studies. Jun Li, Yue Li, Libing Song, Yawei Shi, and Weidong Wei conceived the study, designed the experiments, wrote the manuscript, and supervised the project. The order of the co‐first authors was determined based on their efforts and contributions to the study.

## CONFLICT OF INTEREST STATEMENT

The authors declare no conflict of interest.

## ETHICS APPROVAL AND CONSENT TO PARTICIPATE

This study was conducted in accordance with the principles of the Declaration of Helsinki. Ethics approval for the use of human clinical specimens was obtained from the Institutional Research Ethics Committee of Sun Yat‐sen University Cancer Center (no. B2024‐053‐01). Prior written informed consent was obtained from all patients before they participated in this study. All animal experimental procedures were approved by the Institutional Animal Care and Use Committee of Sun Yat‐sen University (no. L102012022228U).

## Supporting information



Supporting Information

Supporting Information

Supporting Information

Supporting Information

Supporting Information

Supporting Information

## Data Availability

All data generated or analyzed during this study are included in this published article and its supplementary information files.
