# Supplementary figures and images for: RNF31 induces paclitaxel resistance by sustaining ALYREF cytoplasmic–nuclear shuttling in human triple‐negative breast cancer
Source: Clin Transl Med. 2025 Feb 6;15(2):e70203. doi: 10.1002/ctm2.70203 (PMC11802238; doi:10.1002/ctm2.70203)

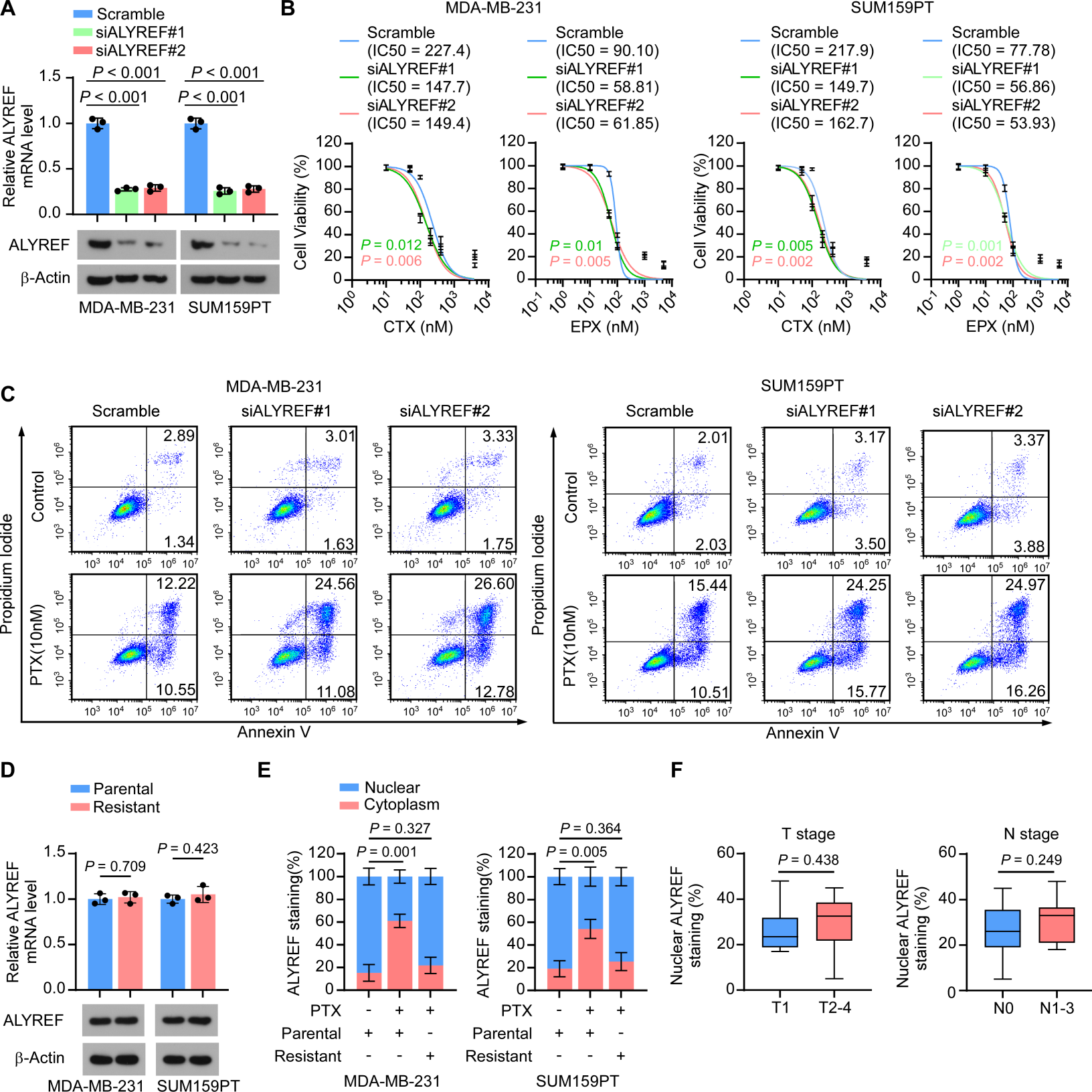

Supplement: Supplementary file 1 — Supporting Information [file CTM2-15-e70203-s005.pdf]

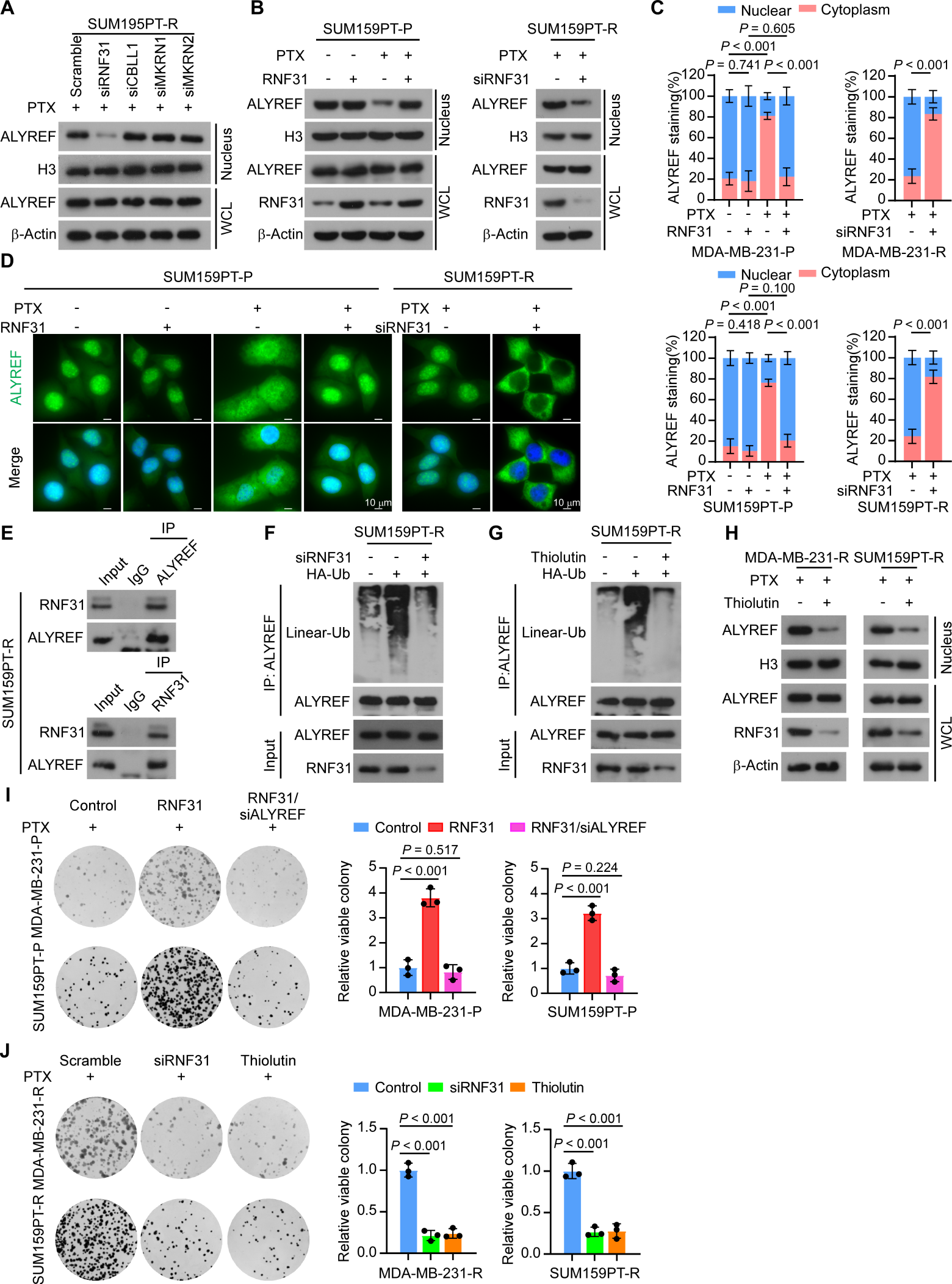

Supplement: Supplementary file 2 — Supporting Information [file CTM2-15-e70203-s004.pdf]

**A**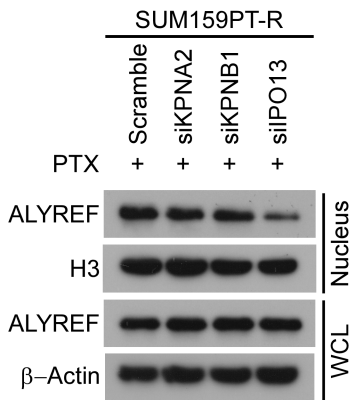**B**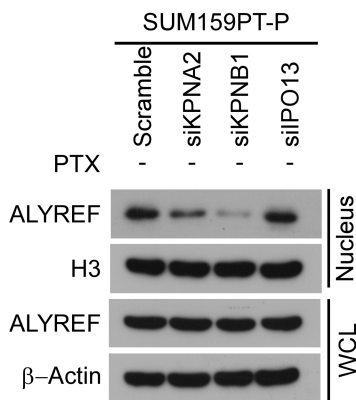**C**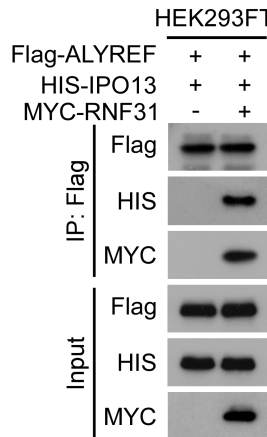**D**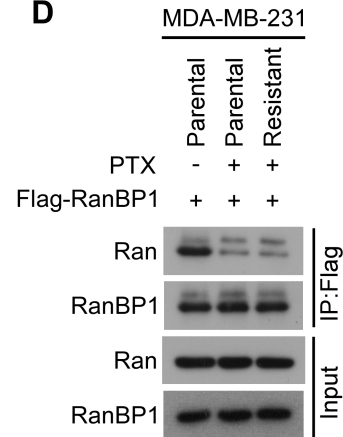**E**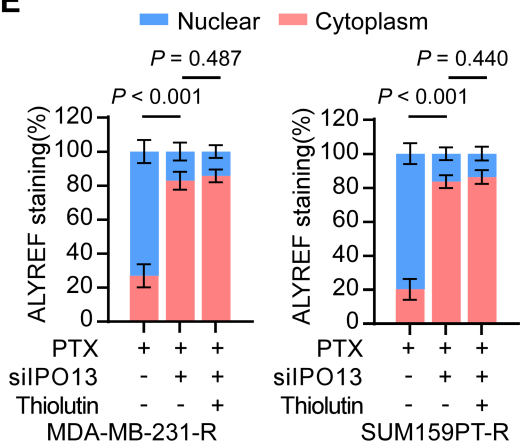**F**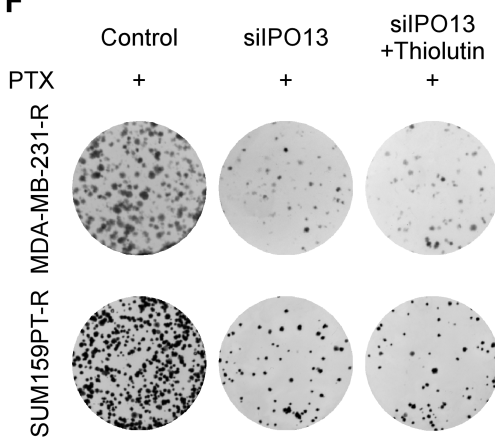

Supplement: Supplementary file 3 — Supporting Information [file CTM2-15-e70203-s003.pdf]

**A**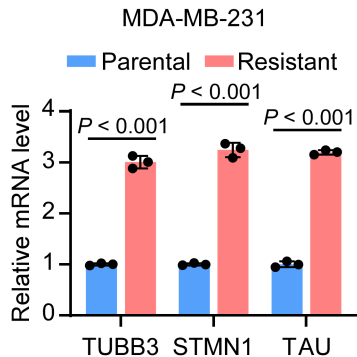

SUM159PT

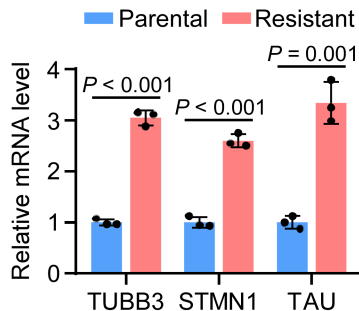**C**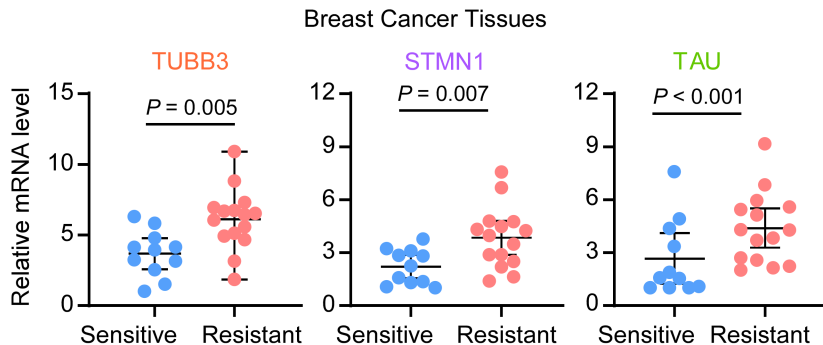**B**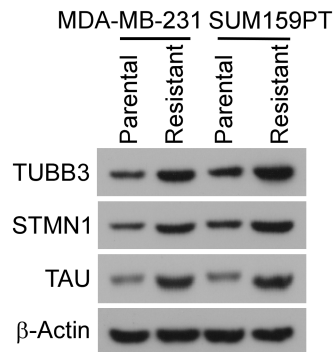**D**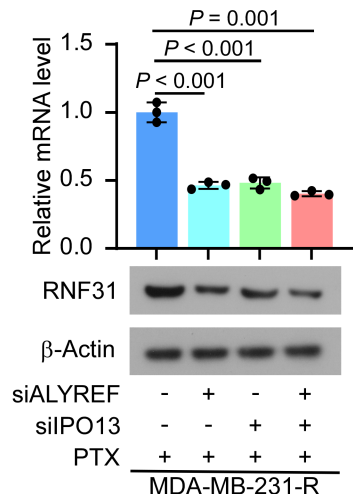

Supplement: Supplementary file 4 — Supporting Information [file CTM2-15-e70203-s006.pdf]

**A**

MDA-MB-231-R/shRNF31  
Organoid #1  
Organoid #2

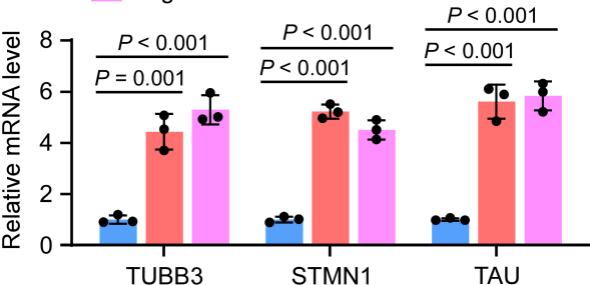

Supplement: Supplementary file 5 — Supporting Information [file CTM2-15-e70203-s002.pdf]
