# Supplementary material for: RNF31 induces paclitaxel resistance by sustaining ALYREF cytoplasmic–nuclear shuttling in human triple‐negative breast cancer
Source: Clin Transl Med. 2025 Feb 6;15(2):e70203. doi: 10.1002/ctm2.70203 (PMC11802238; doi:10.1002/ctm2.70203)
Supplement: Supplementary file 6 — Supporting Information [file CTM2-15-e70203-s001.docx]

**Supplementary Figure 1. A.** Efficiency of siRNA-mediated knockdown of *ALYREF*, as detected using qRT-PCR and western blotting in MDA-MB-231 and SUM159PT cells. **B.** Cell viability assay in the indicated groups. IC50, half maximal inhibitory concentration. CTX, cyclophosphamide. EPX, epirubixin. **C.** Representative images of the cell apoptosis rate, as assessed using flow cytometry in the indicated groups. **D.** mRNA and protein levels of ALYREF in parental and PTX-resistant MDA-MB-231 and SUM159PT cells, as assessed using qRT-PCR and western blotting. **E.** Statistical analysis of the subcellular proportion of ALYREF in the indicated groups. **F.** Quantification of nuclear ALYREF staining in patients with different T stage and N stage.

**Supplementary Figure 2. A.** Western blotting showing the nuclear and total levels of ALYREF upon siRNA-mediated knockdown of *RNF31*, *CBLL1*, *MKRN1*, or *MKRN2* in SUM159PT-R cells. **B.** The nuclear and total levels of ALYREF in SUM159PT-P cells under the indicated treatments, as detected using western blotting (**left**). The nuclear and total ALYREF level upon silencing of *RNF31* in SUM159PT-R cells, as detected using western blotting (**right**). **C.** Statistical analysis of the subcellular proportion of ALYREF detected by IF assays in MDA-MB-231 cells in the indicated groups. **D.** IF assay showing the subcellular distribution of ALYREF in SUM159PT cells in the indicated groups. Scale bar, 10 μm. Representative images (left), statistical analysis (right). **E.** Endogenous Co-IP assays to determine the interaction between ALYREF and RNF31 in SUM159PT-R cells. **F.** Co-IP assay to determine the linear ubiquitylation level of ALYREF in SUM159PT-R cells upon siRNA‑knockdown of *RNF31*. **G.** Co-IP assay to determine the linear ubiquitylation level of ALYREF in SUM159PT-R cells upon Thiolutin treatment. **H.** Western blotting showing the nuclear ALYREF level upon Thiolutin treatment in MDA-MB-231-R and SUM159PT-R cells. **I.** Colony formation assays showing the number of surviving colonies of MDA-MB-231-P and SUM159PT-P cells. Representative images (left). Statistical analysis (right). **J.** Colony formation assays showing the number of surviving colonies of MDA-MB-231-R and SUM159PT-R cells under the indicated treatments. Representative images (left). Statistical analysis (right).

**Supplementary Figure 3. A.** Western blotting showing the nuclear and total levels of ALYREF upon siRNA-mediated knockdown of *IPO13*, *KPNA2* or *KPNB1* in SUM159PT-R cells. **B.** Western blotting showing the nuclear and total levels of ALYREF upon siRNA‑mediated knockdown of *IPO13*, *KPNA2* or *KPNB1* in SUM159PT-P cells without paclitaxel treatment. **C.** Western blotting followed by Co-IP assays showing the interaction between ALYREF and IPO13 in HEK293FT cells in the indicated groups. D**.** Co-IP assay showing the binding ability of Ran with RanBP1 in MDA-MB-231 cells with the indicated treatments. **E.** Statistical analysis of the subcellular proportion of ALYREF by IF assay in the indicated groups. **F.** Images of colony formation assays in the indicated groups.

**Supplementary Figure 4. A.** mRNA levels of *TUBB3*, *STMN1,* and *TAU* in parental and paclitaxel-resistant MDA-MB-231 and SUM159PT cells, as determined using qRT-PCR. **B.** Protein levels of TUBB3, STMN1, and TAU in parental and paclitaxel-resistant MDA-MB-231 and SUM159PT cells, as determined using western blotting. **C.** mRNA levels of *TUBB3*, *STMN1*, and *TAU* in paclitaxel based-neoadjuvant chemotherapy resistant (n = 15) and sensitive (n = 11) patients with TNBC, as detected using qRT-PCR. **D.** mRNA and protein levels of RNF31, as detected using qRT-PCR and western blotting in the indicated groups.

**Supplementary Figure 5. A.** mRNA levels of *TUBB3*, *STMN1*, and *TAU* in breast cancer organoids and RNF31-silenced MDA-MB-231-R cells, as detected using qRT-PCR.

**Supplementary Table 1. Clinical characteristics of patients with TNBC**

| **Patient Number** | N = 183 (%) |
| --- | --- |
| **Age**  Median (range) (year) | 46 (25-72) |
| **Type of Pathology**  Invasive ductal carcinoma  Other | 174 (95.1)  9 (4.9) |
| **Grade of Pathology**  II  III  NA | 33 (18.0)  141 (77.0)  9 (4.9) |
| **Vessel carcinoma embolus**  No  Yes | 123 (67.2)  60 (32.8) |
| **Neural Invasion**  No  Yes | 170 (92.9)  13 (7.1) |
| **T stage**  T1  T2  T3  T4 | 59 (32.2)  106 (57.9)  15 (8.2)  3 (1.6) |
| **N stage**  N0  N1  N2  N3 | 87 (47.5)  55 (30.1)  19 (10.4)  22 (12.0) |
| **Ki67 Index**  < 30  ≥ 30  NA | 22 (12.0)  158 (86.3)  3 (1.6) |
| **Neo Adjuvant Chemotherapy**  Yes  No | 26(14.2)  157(85.8) |
| **Chemotherapy Strategy**  AC-T  TEC  TC  Other | 122(66.7)  20(10.9)  19(10.4)  22(12.0) |
| **Type of Taxane**  Paclitaxel  Docetaxel  Other | 91 (49.7)  83 (45.4)  9 (4.9) |
| **RNF31**  Low  High | 87 (47.5)  96 (52.5) |

**Supplementary Table 2. Clinical characteristics of patients with TNBC with different RNF31 levels**

|  | **RNF31 low**  **N = 87 (%)** | **RNF31 high**  **N = 96 (%)** | ***P* value** |
| --- | --- | --- | --- |
| **Age**  Median (range) (year) | 45 (25-72) | 46 (28-68) | 0.633 |
| **Type of Pathology**  Invasive ductal carcinoma  Other | 83 (95.4)  4 (4.6) | 91 (94.8)  5 (5.2) | 0.849 |
| **Grade of Pathology**  II  III  NA | 20 (23.0)  63 (72.4)  4 (4.6) | 13 (13.5)  78 (81.3)  5 (5.2) | 0.252 |
| **Vessel carcinoma embolus**  No  Yes | 65 (74.7)  22 (25.3) | 58 (60.4)  38 (39.6) | 0.040 |
| **Neural Invasion**  No  Yes | 79 (90.8)  8 (9.2) | 91 (94.8)  5 (5.2) | 0.294 |
| **T stage**  T1  T2  T3  T4 | 31 (35.6)  53 (60.9)  2 (2.3)  1 (1.1) | 28 (29.2)  53 (55.2)  13 (13.5)  2 (2.1) | 0.043 |
| **N stage**  N0  N1  N2  N3 | 44 (50.6)  25 (28.7)  8 (9.2)  10 (11.5) | 43 (44.8)  30 (31.3)  11 (11.5)  12 (12.5) | 0.878 |
| **Ki67 Index**  < 30  ≥ 30  NA | 11 (12.6)  74 (85.1)  2 (2.3) | 11 (11.5)  84 (87.5)  1 (1.0) | 0.769 |

**Supplementary Table 3. Treatment strategies of patients with TNBC**

|  | **Total**  **N = 183 (%)** | **RNF31 low**  **N = 87 (%)** | **RNF31 high**  **N = 96 (%)** | ***P* value** |
| --- | --- | --- | --- | --- |
| **Chemotherapy** Strategy  AC-T  TEC  TC  Other | 122 (66.7)  20 (10.9)  19 (10.4)  22 (12.0) | 59 (67.8)  6 (6.9)  7 (8.0)  15 (17.2) | 63 (65.6)  14 (14.6)  12 (12.5)  7 (7.3) | 0.068 |
| **Type of Taxane**  Paclitaxel  Docetaxel  Other | 91 (49.7)  83 (45.4)  9 (4.9) | 45 (51.7)  41 (47.1)  1 (1.1) | 46 (47.9)  42 (43.8)  8 (8.3) | 0.081 |

**Supplementary Table 4. Univariate Cox Regression analysis (Backward LR) of OS in patients with TNBC (n = 183)**

|  | ***P* value** | **HR (95% CI)** |
| --- | --- | --- |
| **Age**  ≥ 60y/< 60y | 0.036 | 2.120 (1.051-4.275) |
| **Type of Pathology**  Invasive ductal carcinoma/Other | 0.548 | 1.432 (0.444-4.617) |
| **Grade of Pathology**  III/II | 0.818 | 1.094 (0.508-2.360) |
| **Vessel carcinoma embolus**  Yes/No | 0.033 | 1.887 (1.052-3.383) |
| **Neural Invasion**  Yes/No | 0.001 | 3.764 (1.751-8.090) |
| **T stage**  T2-4/T1 | 0.316 | 1.389 (0.731-2.640) |
| **N stage**  N1-3/N0 | 0.001 | 3.101 (1.605-5.993) |
| **Ki67 Index**  ≥ 30/< 30 | 0.744 | 0.874 (0.391-1.955) |
| **RNF31**  High/Low | 0.002 | 2.723 (1.449-5.116) |
| **Chemotherapy** **Strategy**  TEC/AC-T  TC/AC-T  Other/AC-T | 0.007  0.005  0.694  0.104 | 2.767 (1.349-5.675)  0.811 (0.286-2.303)  0.305 (0.073-1.277) |
| **Type of Taxane**  Docetaxel/ Paclitaxel  Other/ Paclitaxel | 0.786  0.744  0.499 | 1.105 (0.607-2.010)  1.518 (0.452-5.091) |

**Supplementary Table 5. Multivariate Cox Regression analysis (Backward LR) of OS in patients with TNBC (n = 183)**

|  | ***P* value** | **HR (95% CI)** |
| --- | --- | --- |
| **Age**  ≥ 60y/< 60y | 0.020 | 2.318 (1.144-4.698) |
| **Vessel carcinoma embolus**  Yes/No | 0.602 | 0.838 (0.432-1.627) |
| **Neural Invasion**  Yes/No | 0.002 | 3.559 (1.610-7.864) |
| **T stage**  T2-4/T1 | 0.723 | 0.885 (0.452-1.734) |
| **N stage**  N1-3/N0 | 0.003 | 2.742 (1.394-5.392) |
| **RNF31**  High/Low | 0.002 | 2.790 (1.467-5.305) |
| **Chemotherapy** **Strategy**  TEC/AC-T  TC/AC-T  Other/AC-T | 0.138  0.115  0.678  0.147 | 1.821 (0.864-3.835)  0.800 (0.280-2.287)  0.346 (0.082-1.454) |

**Supplementary table 6. Protein information for ALYREF interactors in MDA-MB-231-R**

| **Accession number** | **Area of anti‑ALYREF** | **Area of IgG** | **LOG FC** |
| --- | --- | --- | --- |
| sp\|Q07021\|C1QBP_HUMAN | 1.40E+10 | 2.36E+08 | 1.77E+00 |
| sp\|Q86V81\|THOC4_HUMAN | 2.07E+10 |  | 1.03E+01 |
| sp\|Q15029\|U5S1_HUMAN | 1.44E+08 | 1.32E+07 | 1.04E+00 |
| sp\|Q96EP0\|RNF31_HUMAN | 1.70E+08 |  | 8.23E+00 |
| sp\|Q12849\|GRSF1_HUMAN | 4.58E+08 |  | 8.66E+00 |
| sp\|Q99873\|ANM1_HUMAN | 5.37E+08 |  | 8.73E+00 |
| sp\|Q96EY7\|PTCD3_HUMAN | 1.97E+08 | 1.12E+05 | 3.25E+00 |
| sp\|Q69YN4\|VIR_HUMAN | 6.05E+07 | 2.42E+06 | 1.40E+00 |
| sp\|P09874\|PARP1_HUMAN | 2.96E+07 | 4.05E+05 | 1.86E+00 |
| sp\|Q14974\|IMB1_HUMAN | 8.11E+07 | 6.15E+04 | 3.12E+00 |
| sp\|P12956\|XRCC6_HUMAN | 7.88E+07 | 6.02E+06 | 1.12E+00 |
| sp\|P51398\|RT29_HUMAN | 1.71E+08 |  | 8.23E+00 |
| sp\|Q92552\|RT27_HUMAN | 1.12E+08 |  | 8.05E+00 |
| sp\|P19338\|NUCL_HUMAN | 1.96E+08 | 5.74E+05 | 2.53E+00 |
| sp\|P82933\|RT09_HUMAN | 1.42E+08 | 1.45E+05 | 2.99E+00 |
| sp\|Q92665\|RT31_HUMAN | 1.29E+08 |  | 8.11E+00 |
| sp\|P22626\|ROA2_HUMAN | 6.01E+07 | 4.38E+06 | 1.14E+00 |
| sp\|Q9Y2R9\|RT07_HUMAN | 1.22E+08 |  | 8.09E+00 |
| sp\|P82650\|RT22_HUMAN | 8.19E+07 |  | 7.91E+00 |
| sp\|Q12905\|ILF2_HUMAN | 6.04E+07 | 2.01E+06 | 1.48E+00 |
| sp\|Q96DI7\|SNR40_HUMAN | 3.74E+07 | 1.55E+06 | 1.38E+00 |
| sp\|Q9BUJ2\|HNRL1_HUMAN | 3.60E+07 | 5.49E+05 | 1.82E+00 |
| sp\|P82673\|RT35_HUMAN | 1.55E+08 |  | 8.19E+00 |
| sp\|P67809\|YBOX1_HUMAN | 3.48E+07 | 2.52E+06 | 1.14E+00 |
| sp\|P82675\|RT05_HUMAN | 6.82E+07 |  | 7.83E+00 |
| sp\|Q9NW64\|RBM22_HUMAN | 2.01E+07 | 2.10E+05 | 1.98E+00 |
| sp\|O75152\|ZC11A_HUMAN | 1.58E+07 |  | 7.20E+00 |
| sp\|P09651\|ROA1_HUMAN | 2.00E+07 | 8.64E+05 | 1.36E+00 |
| sp\|Q9Y676\|RT18B_HUMAN | 6.95E+07 |  | 7.84E+00 |
| sp\|O75367\|H2AY_HUMAN | 1.41E+07 |  | 7.15E+00 |
| sp\|Q9Y3D9\|RT23_HUMAN | 3.27E+07 |  | 7.51E+00 |
| sp\|Q15393\|SF3B3_HUMAN | 2.68E+07 | 0.00E+00 | 7.43E+00 |
| sp\|Q9Y399\|RT02_HUMAN | 4.10E+07 |  | 7.61E+00 |
| sp\|P62906\|RL10A_HUMAN | 2.70E+08 | 5.44E+06 | 1.70E+00 |
| sp\|P22087\|FBRL_HUMAN | 4.92E+07 | 4.65E+06 | 1.02E+00 |
| sp\|Q96EL2\|RT24_HUMAN | 5.93E+07 |  | 7.77E+00 |
| sp\|Q6P158\|DHX57_HUMAN | 6.05E+06 |  | 6.78E+00 |
| sp\|Q9BWF3\|RBM4_HUMAN | 9.64E+06 |  | 6.98E+00 |
| sp\|P82930\|RT34_HUMAN | 7.20E+07 |  | 7.86E+00 |
| sp\|Q15007\|FL2D_HUMAN | 2.23E+07 | 7.44E+05 | 1.48E+00 |
| sp\|Q96T58\|MINT_HUMAN | 9.07E+06 |  | 6.96E+00 |
| sp\|P49792\|RBP2_HUMAN | 7.75E+06 |  | 6.89E+00 |
| sp\|P82663\|RT25_HUMAN | 6.77E+07 |  | 7.83E+00 |
| sp\|Q9BYN8\|RT26_HUMAN | 4.44E+07 |  | 7.65E+00 |
| sp\|Q53F19\|NCBP3_HUMAN | 2.15E+07 |  | 7.33E+00 |
| sp\|P82664\|RT10_HUMAN | 2.04E+07 |  | 7.31E+00 |
| sp\|Q16777\|H2A2C_HUMAN | 5.47E+06 |  | 6.74E+00 |
| sp\|O60841\|IF2P_HUMAN | 7.91E+06 | 8.21E+04 | 1.98E+00 |
| sp\|P46087\|NOP2_HUMAN | 1.27E+07 |  | 7.10E+00 |
| sp\|O60306\|AQR_HUMAN | 8.14E+06 |  | 6.91E+00 |
| sp\|P0C0S5\|H2AZ_HUMAN | 1.15E+07 |  | 7.06E+00 |
| sp\|P62805\|H4_HUMAN | 5.87E+08 | 2.25E+07 | 1.42E+00 |
| sp\|Q9BZJ0\|CRNL1_HUMAN | 9.39E+06 | 5.79E+05 | 1.21E+00 |
| sp\|P06280\|AGAL_HUMAN | 5.97E+06 |  | 6.78E+00 |
| sp\|Q9BYX7\|ACTBM_HUMAN | 4.39E+06 |  | 6.64E+00 |
| sp\|Q9Y3D3\|RT16_HUMAN | 5.24E+07 |  | 7.72E+00 |
| sp\|P82932\|RT06_HUMAN | 3.50E+07 |  | 7.54E+00 |
| sp\|P16402\|H13_HUMAN | 3.95E+06 |  | 6.60E+00 |
| sp\|P16104\|H2AX_HUMAN | 5.49E+06 |  | 6.74E+00 |
| sp\|O75934\|SPF27_HUMAN | 9.30E+06 | 2.07E+05 | 1.65E+00 |
| sp\|Q99729\|ROAA_HUMAN | 3.75E+06 | 1.14E+05 | 1.52E+00 |
| sp\|P33778\|H2B1B_HUMAN | 1.43E+07 | 5.33E+05 | 1.43E+00 |
| sp\|P61326\|MGN_HUMAN | 2.63E+05 |  | 5.42E+00 |
| sp\|Q9HCS7\|SYF1_HUMAN | 8.59E+06 | 5.37E+05 | 1.20E+00 |
| sp\|P62888\|RL30_HUMAN | 6.34E+07 | 1.74E+06 | 1.56E+00 |
| sp\|Q9BZE4\|GTPB4_HUMAN | 3.61E+06 |  | 6.56E+00 |
| sp\|O60814\|H2B1K_HUMAN | 2.56E+07 |  | 7.41E+00 |
| sp\|Q8NAV1\|PR38A_HUMAN | 1.08E+07 | 4.72E+05 | 1.36E+00 |
| sp\|Q99538\|LGMN_HUMAN | 8.90E+06 |  | 6.95E+00 |
| sp\|P55081\|MFAP1_HUMAN | 7.17E+06 |  | 6.86E+00 |
| sp\|Q12873\|CHD3_HUMAN | 9.65E+06 |  | 6.98E+00 |
| sp\|Q14839\|CHD4_HUMAN | 1.29E+06 |  | 6.11E+00 |
| sp\|Q13435\|SF3B2_HUMAN | 6.20E+06 | 2.84E+05 | 1.34E+00 |
| sp\|Q71DI3\|H32_HUMAN | 1.01E+08 | 7.49E+06 | 1.13E+00 |
| sp\|P84243\|H33_HUMAN | 3.21E+07 | 3.18E+06 | 1.00E+00 |
| sp\|Q14978\|NOLC1_HUMAN | 2.66E+07 | 1.91E+06 | 1.14E+00 |
| sp\|P05455\|LA_HUMAN | 4.11E+06 |  | 6.61E+00 |
| sp\|Q9BZE1\|RM37_HUMAN | 2.42E+06 |  | 6.38E+00 |
| sp\|P68431\|H31_HUMAN | 5.47E+07 |  | 7.74E+00 |
| sp\|Q9Y2Q9\|RT28_HUMAN | 2.07E+07 |  | 7.32E+00 |
| sp\|Q96DV4\|RM38_HUMAN | 6.23E+06 |  | 6.79E+00 |
| sp\|O95793\|STAU1_HUMAN | 2.23E+06 |  | 6.35E+00 |
| sp\|P84085\|ARF5_HUMAN | 3.55E+05 |  | 5.55E+00 |
| sp\|Q9H2Y7\|ZN106_HUMAN | 2.00E+06 |  | 6.30E+00 |
| sp\|Q9HD33\|RM47_HUMAN | 3.39E+06 |  | 6.53E+00 |
| sp\|P08779\|K1C16_HUMAN | 2.73E+05 |  | 5.44E+00 |
| sp\|Q9Y2S7\|PDIP2_HUMAN | 2.89E+06 |  | 6.46E+00 |
| sp\|Q96GQ7\|DDX27_HUMAN | 3.82E+06 | 2.41E+05 | 1.20E+00 |
| sp\|P78362\|SRPK2_HUMAN | 1.69E+06 |  | 6.23E+00 |
| sp\|Q9Y2R5\|RT17_HUMAN | 6.31E+07 |  | 7.80E+00 |
| sp\|P51532\|SMCA4_HUMAN | 8.41E+05 |  | 5.92E+00 |
| sp\|P55769\|NH2L1_HUMAN | 1.25E+07 | 2.82E+05 | 1.65E+00 |
| sp\|Q8N0Z8\|PUSL1_HUMAN | 3.16E+06 |  | 6.50E+00 |
| sp\|Q9Y5T5\|UBP16_HUMAN | 3.03E+07 |  | 7.48E+00 |
| sp\|Q96SI9\|STRBP_HUMAN | 1.76E+06 |  | 6.25E+00 |
| sp\|Q13084\|RM28_HUMAN | 1.19E+06 |  | 6.08E+00 |
| sp\|Q9H7B2\|RPF2_HUMAN | 3.98E+06 |  | 6.60E+00 |
| sp\|O15226\|NKRF_HUMAN | 1.97E+06 |  | 6.29E+00 |
| sp\|P19525\|E2AK2_HUMAN | 2.74E+06 | 1.48E+05 | 1.27E+00 |
| sp\|Q9P0M6\|H2AW_HUMAN | 1.27E+05 |  | 5.10E+00 |
| sp\|Q96PK6\|RBM14_HUMAN | 1.56E+07 | 4.33E+05 | 1.56E+00 |
| sp\|Q9P015\|RM15_HUMAN | 2.46E+06 |  | 6.39E+00 |
| sp\|Q15427\|SF3B4_HUMAN | 4.96E+06 |  | 6.70E+00 |
| sp\|Q4G0J3\|LARP7_HUMAN | 2.13E+06 |  | 6.33E+00 |
| sp\|Q15459\|SF3A1_HUMAN | 2.27E+06 |  | 6.36E+00 |
| sp\|Q9Y3C6\|PPIL1_HUMAN | 7.73E+06 |  | 6.89E+00 |
| sp\|P56537\|IF6_HUMAN | 5.52E+05 |  | 5.74E+00 |
| sp\|Q9H0D6\|XRN2_HUMAN | 2.36E+06 |  | 6.37E+00 |
| sp\|Q9P035\|HACD3_HUMAN | 1.15E+06 |  | 6.06E+00 |
| sp\|Q9NX24\|NHP2_HUMAN | 8.41E+06 |  | 6.92E+00 |
| sp\|Q8TAQ2\|SMRC2_HUMAN | 1.55E+06 |  | 6.19E+00 |
| sp\|O60783\|RT14_HUMAN | 1.47E+07 |  | 7.17E+00 |
| sp\|Q14739\|LBR_HUMAN | 4.58E+06 | 1.54E+05 | 1.47E+00 |
| sp\|P10809\|CH60_HUMAN | 9.04E+05 |  | 5.96E+00 |
| sp\|P0C0L5\|CO4B_HUMAN | 4.05E+05 |  | 5.61E+00 |
| sp\|Q6PJT7\|ZC3HE_HUMAN | 3.76E+06 |  | 6.58E+00 |
| sp\|Q9NPE3\|NOP10_HUMAN | 5.16E+06 |  | 6.71E+00 |
| sp\|A0A3B3IS91\|PLGRF_HUMAN | 6.14E+05 |  | 5.79E+00 |
| sp\|Q9UNP9\|PPIE_HUMAN | 1.21E+06 |  | 6.08E+00 |
| sp\|Q04837\|SSBP_HUMAN | 1.66E+06 |  | 6.22E+00 |
| sp\|Q9BYD3\|RM04_HUMAN | 1.63E+06 |  | 6.21E+00 |
| sp\|Q7Z2W4\|ZCCHV_HUMAN | 9.92E+05 |  | 6.00E+00 |
| sp\|Q9H2U1\|DHX36_HUMAN | 6.77E+05 | 5.73E+04 | 1.07E+00 |
| sp\|O43660\|PLRG1_HUMAN | 2.76E+06 |  | 6.44E+00 |
| sp\|Q9H9J2\|RM44_HUMAN | 3.48E+06 |  | 6.54E+00 |
| sp\|P01861\|IGHG4_HUMAN | 3.08E+09 | 2.41E+08 | 1.11E+00 |
| sp\|O76094\|SRP72_HUMAN | 1.25E+06 | 1.25E+05 | 1.00E+00 |
| sp\|Q92522\|H1X_HUMAN | 1.91E+06 |  | 6.28E+00 |
| sp\|Q13185\|CBX3_HUMAN | 2.48E+06 | 2.27E+05 | 1.04E+00 |
| sp\|Q9BYD6\|RM01_HUMAN | 1.99E+06 |  | 6.30E+00 |
| sp\|Q5TEC6\|H37_HUMAN | 4.96E+06 |  | 6.70E+00 |
| sp\|P01857\|IGHG1_HUMAN | 2.18E+04 |  | 4.34E+00 |
| sp\|Q96FJ2\|DYL2_HUMAN | 5.14E+06 |  | 6.71E+00 |
| sp\|P32119\|PRDX2_HUMAN | 3.38E+06 |  | 6.53E+00 |
| sp\|P08579\|RU2B_HUMAN | 7.46E+05 |  | 5.87E+00 |
| sp\|P62306\|RUXF_HUMAN | 6.27E+06 |  | 6.80E+00 |
| sp\|Q15050\|RRS1_HUMAN | 1.34E+06 |  | 6.13E+00 |
| sp\|Q9NWU5\|RM22_HUMAN | 1.76E+06 |  | 6.25E+00 |
| sp\|Q7RTV0\|PHF5A_HUMAN | 1.33E+06 |  | 6.12E+00 |
| sp\|P54105\|ICLN_HUMAN | 7.67E+05 |  | 5.88E+00 |
| sp\|Q8TAE8\|G45IP_HUMAN | 1.19E+06 |  | 6.08E+00 |
| sp\|Q9P013\|CWC15_HUMAN | 3.30E+06 |  | 6.52E+00 |
| sp\|Q14690\|RRP5_HUMAN | 4.81E+05 |  | 5.68E+00 |
| sp\|P01344\|IGF2_HUMAN | 8.95E+06 |  | 6.95E+00 |
| sp\|Q9NRX2\|RM17_HUMAN | 1.32E+06 |  | 6.12E+00 |
| sp\|A4D1E1\|Z804B_HUMAN | 2.78E+04 |  | 4.44E+00 |
| sp\|Q75N03\|HAKAI_HUMAN | 1.27E+06 |  | 6.10E+00 |
| sp\|Q9BQG0\|MBB1A_HUMAN | 1.32E+05 |  | 5.12E+00 |
| sp\|Q13405\|RM49_HUMAN | 1.03E+06 |  | 6.01E+00 |
| sp\|Q9H3K6\|BOLA2_HUMAN | 3.39E+06 |  | 6.53E+00 |
| sp\|O43684\|BUB3_HUMAN | 6.72E+05 |  | 5.83E+00 |
| sp\|Q13547\|HDAC1_HUMAN | 3.32E+05 |  | 5.52E+00 |
| sp\|Q7Z2W9\|RM21_HUMAN | 1.71E+06 |  | 6.23E+00 |
| sp\|Q8IXM3\|RM41_HUMAN | 1.81E+06 |  | 6.26E+00 |
| sp\|P52272\|HNRPM_HUMAN | 6.09E+06 | 2.38E+05 | 1.41E+00 |
| sp\|Q9NUL3\|STAU2_HUMAN | 1.67E+05 |  | 5.22E+00 |
| sp\|Q6DKI1\|RL7L_HUMAN | 8.17E+05 |  | 5.91E+00 |
| sp\|Q01130\|SRSF2_HUMAN | 9.14E+05 |  | 5.96E+00 |
| sp\|Q93009\|UBP7_HUMAN | 8.70E+07 |  | 7.94E+00 |
| sp\|O95819\|M4K4_HUMAN | 1.34E+05 |  | 5.13E+00 |
| sp\|Q96HR8\|NAF1_HUMAN | 1.31E+06 |  | 6.12E+00 |
| sp\|Q01082\|SPTB2_HUMAN | 6.72E+05 |  | 5.83E+00 |
| sp\|Q9NW13\|RBM28_HUMAN | 1.66E+05 |  | 5.22E+00 |
| sp\|Q9H773\|DCTP1_HUMAN | 8.99E+04 |  | 4.95E+00 |
| sp\|Q92820\|GGH_HUMAN | 7.01E+05 |  | 5.85E+00 |
| sp\|Q9Y3D5\|RT18C_HUMAN | 2.81E+06 |  | 6.45E+00 |
| sp\|P57678\|GEMI4_HUMAN | 1.36E+05 |  | 5.13E+00 |
| sp\|Q9BYC8\|RM32_HUMAN | 1.01E+06 |  | 6.00E+00 |
| sp\|Q16527\|CSRP2_HUMAN | 1.89E+06 |  | 6.28E+00 |
| sp\|P27694\|RFA1_HUMAN | 4.84E+05 |  | 5.68E+00 |
| sp\|Q9BYD1\|RM13_HUMAN | 1.90E+06 |  | 6.28E+00 |
| sp\|O75629\|CREG1_HUMAN | 3.23E+06 |  | 6.51E+00 |
| sp\|P04080\|CYTB_HUMAN | 3.97E+05 |  | 5.60E+00 |
| sp\|P49207\|RL34_HUMAN | 3.91E+07 |  | 7.59E+00 |
| sp\|Q8N5N7\|RM50_HUMAN | 3.57E+05 |  | 5.55E+00 |
| sp\|Q06787\|FMR1_HUMAN | 2.30E+05 |  | 5.36E+00 |
| sp\|Q86U86\|PB1_HUMAN | 2.92E+05 |  | 5.47E+00 |
| sp\|Q8IUX4\|ABC3F_HUMAN | 1.75E+05 |  | 5.24E+00 |
| sp\|P61221\|ABCE1_HUMAN | 6.62E+05 |  | 5.82E+00 |
| sp\|Q92928\|RAB1C_HUMAN | 2.98E+06 |  | 6.47E+00 |
| sp\|Q9P275\|UBP36_HUMAN | 7.30E+04 |  | 4.86E+00 |
| sp\|P09001\|RM03_HUMAN | 4.06E+05 |  | 5.61E+00 |
| sp\|Q16540\|RM23_HUMAN | 1.49E+06 |  | 6.17E+00 |
| sp\|Q9UHC7\|MKRN1_HUMAN | 5.96E+05 |  | 5.78E+00 |
| sp\|Q12931\|TRAP1_HUMAN | 2.97E+05 |  | 5.47E+00 |
| sp\|Q96NC0\|ZMAT2_HUMAN | 1.36E+05 |  | 5.13E+00 |
| sp\|P49406\|RM19_HUMAN | 5.10E+05 |  | 5.71E+00 |
| sp\|P28370\|SMCA1_HUMAN | 8.10E+04 |  | 4.91E+00 |
| sp\|Q8N9M1\|CS047_HUMAN | 3.70E+05 |  | 5.57E+00 |
| sp\|Q9ULR0\|ISY1_HUMAN | 5.83E+05 |  | 5.77E+00 |
| sp\|Q9UNX4\|WDR3_HUMAN | 6.55E+04 |  | 4.82E+00 |
| sp\|Q9HB40\|RISC_HUMAN | 1.52E+05 |  | 5.18E+00 |
| sp\|P63208\|SKP1_HUMAN | 5.03E+05 |  | 5.70E+00 |
| sp\|O43447\|PPIH_HUMAN | 1.98E+05 |  | 5.30E+00 |
| sp\|Q9H583\|HEAT1_HUMAN | 2.16E+05 |  | 5.33E+00 |
| sp\|Q96EY1\|DNJA3_HUMAN | 4.67E+05 |  | 5.67E+00 |
| sp\|Q9HCD5\|NCOA5_HUMAN | 1.01E+06 |  | 6.00E+00 |
| sp\|Q9Y230\|RUVB2_HUMAN | 1.45E+05 |  | 5.16E+00 |
| sp\|P37108\|SRP14_HUMAN | 4.70E+05 |  | 5.67E+00 |
| sp\|Q16610\|ECM1_HUMAN | 2.39E+06 |  | 6.38E+00 |
| sp\|Q9Y3B4\|SF3B6_HUMAN | 7.49E+05 |  | 5.87E+00 |
| sp\|Q8N983\|RM43_HUMAN | 5.89E+05 |  | 5.77E+00 |
| sp\|P07602\|SAP_HUMAN | 8.09E+05 |  | 5.91E+00 |
| sp\|P00739\|HPTR_HUMAN | 1.46E+06 |  | 6.16E+00 |
| sp\|P49761\|CLK3_HUMAN | 1.07E+06 |  | 6.03E+00 |
| sp\|Q9H000\|MKRN2_HUMAN | 1.68E+05 |  | 5.23E+00 |
| sp\|P05387\|RLA2_HUMAN | 1.59E+05 |  | 5.20E+00 |
| sp\|P46060\|RAGP1_HUMAN | 1.14E+05 |  | 5.06E+00 |
| sp\|Q96BK5\|PINX1_HUMAN | 5.59E+04 |  | 4.75E+00 |
| sp\|Q9NYK5\|RM39_HUMAN | 7.56E+05 |  | 5.88E+00 |
| sp\|P51654\|GPC3_HUMAN | 1.75E+05 |  | 5.24E+00 |
| sp\|P15586\|GNS_HUMAN | 3.51E+05 |  | 5.55E+00 |
| sp\|P62857\|RS28_HUMAN | 1.17E+06 |  | 6.07E+00 |
| sp\|Q9BZL1\|UBL5_HUMAN | 2.12E+05 |  | 5.33E+00 |
| sp\|P0DJI8\|SAA1_HUMAN | 2.59E+06 |  | 6.41E+00 |
| sp\|Q8NI27\|THOC2_HUMAN | 1.14E+05 |  | 5.06E+00 |
| sp\|Q9H8G2\|CAAP1_HUMAN | 9.30E+05 |  | 5.97E+00 |
| sp\|Q9NZE8\|RM35_HUMAN | 1.38E+05 |  | 5.14E+00 |
| sp\|Q6NSI4\|RADX_HUMAN | 1.44E+05 |  | 5.16E+00 |
| sp\|Q9BYD2\|RM09_HUMAN | 4.69E+05 |  | 5.67E+00 |
| sp\|Q6P6C2\|ALKB5_HUMAN | 7.35E+05 |  | 5.87E+00 |
| sp\|P36873\|PP1G_HUMAN | 2.39E+05 | 0.00E+00 | 5.38E+00 |
| sp\|Q9BW60\|ELOV1_HUMAN | 7.16E+04 |  | 4.85E+00 |
| sp\|Q9BRJ2\|RM45_HUMAN | 6.76E+05 |  | 5.83E+00 |
| sp\|P22692\|IBP4_HUMAN | 8.41E+05 |  | 5.92E+00 |
| sp\|P12268\|IMDH2_HUMAN | 5.15E+05 |  | 5.71E+00 |
| sp\|Q9BYG3\|MK67I_HUMAN | 3.37E+05 |  | 5.53E+00 |
| sp\|O14893\|GEMI2_HUMAN | 2.58E+05 |  | 5.41E+00 |
| sp\|P52292\|IMA1_HUMAN | 2.07E+05 |  | 5.32E+00 |
| sp\|O75376\|NCOR1_HUMAN | 2.51E+05 |  | 5.40E+00 |
| sp\|Q96T88\|UHRF1_HUMAN | 1.53E+05 |  | 5.18E+00 |
| sp\|O00160\|MYO1F_HUMAN | 1.75E+04 |  | 4.24E+00 |
| sp\|Q8TDD1\|DDX54_HUMAN | 2.27E+05 |  | 5.36E+00 |
| sp\|Q9Y4F5\|C170B_HUMAN | 6.00E+05 |  | 5.78E+00 |
| sp\|Q04323\|UBXN1_HUMAN | 1.95E+05 |  | 5.29E+00 |
| sp\|P17931\|LEG3_HUMAN | 3.64E+05 |  | 5.56E+00 |
| sp\|Q86U42\|PABP2_HUMAN | 2.37E+05 |  | 5.37E+00 |
| sp\|Q05397\|FAK1_HUMAN | 1.81E+05 |  | 5.26E+00 |
| sp\|P51693\|APLP1_HUMAN | 1.14E+06 |  | 6.06E+00 |
| sp\|P41250\|GARS_HUMAN | 1.58E+06 |  | 6.20E+00 |
| sp\|O15260\|SURF4_HUMAN | 2.61E+05 |  | 5.42E+00 |
| sp\|O94916\|NFAT5_HUMAN | 1.93E+05 |  | 5.29E+00 |
| sp\|Q5M9N0\|CD158_HUMAN | 4.96E+05 |  | 5.70E+00 |
| sp\|Q7Z745\|MRO2B_HUMAN | 3.99E+05 |  | 5.60E+00 |
| sp\|P31040\|SDHA_HUMAN | 8.69E+05 |  | 5.94E+00 |
| sp\|O60271\|JIP4_HUMAN | 1.12E+06 |  | 6.05E+00 |
| sp\|Q96T37\|RBM15_HUMAN | 9.71E+05 |  | 5.99E+00 |
| sp\|O00203\|AP3B1_HUMAN | 1.58E+05 |  | 5.20E+00 |
| sp\|P23258\|TBG1_HUMAN | 5.40E+04 |  | 4.73E+00 |
| sp\|O75460\|ERN1_HUMAN | 1.45E+04 |  | 4.16E+00 |
| sp\|Q7Z7M0\|MEGF8_HUMAN | 4.77E+05 |  | 5.68E+00 |
| sp\|Q14314\|FGL2_HUMAN | 7.93E+05 |  | 5.90E+00 |
| sp\|Q14159\|SPIDR_HUMAN | 4.18E+07 |  | 7.62E+00 |
| sp\|A0A0B4J1V7\|HV781_HUMAN | 3.17E+06 |  | 6.50E+00 |
| sp\|A0A087WSY6\|KVD15_HUMAN | 4.85E+05 |  | 5.69E+00 |
| sp\|A2A3N6\|PIPSL_HUMAN | 1.11E+07 |  | 7.05E+00 |
| sp\|Q15633\|TRBP2_HUMAN | 9.99E+04 |  | 5.00E+00 |
| sp\|Q2M1Z3\|RHG31_HUMAN | 2.38E+06 |  | 6.38E+00 |
| sp\|Q9NVS2\|RT18A_HUMAN | 5.18E+05 |  | 5.71E+00 |
| sp\|P0C091\|FREM3_HUMAN | 1.78E+06 |  | 6.25E+00 |

**Supplementary Materials and Methods**

**Cells**

The human triple negative breast cancer cell line MDA-MB-231 was obtained from the American Type Culture Collection (ATCC, Manassas, VA, USA), and SUM159PT cells were obtained from Asterand Bioscience (Royston, UK). The cell lines were authenticated using short tandem repeat (STR) fingerprinting. MDA-MB-231 cells were maintained in Dulbecco’s modified Eagle’s medium (DMEM), and SUM159PT cells were maintained in DMEM/F12 (1:1), supplemented with 10% fetal bovine serum (FBS), penicillin, and streptomycin. Paclitaxel-resistant MDA-MB-231 and SUM159PT cells lines were established by exposure to increasing doses of paclitaxel. Briefly, MDA-MB-231 and SUM159PT cells were exposed to an initial paclitaxel concentration of 0.01 nM for two days, and then kept in drug-free culture medium until the next mitotic phase. The IC50 values of paclitaxel in parental MDA-MB-231 cells and SUM159PT cells were 12.660 nM, and 11.730 nM, respectively. Paclitaxel-resistant cell lines were established when the IC50 of paclitaxel displayed at least a five-fold increase. The IC50 of paclitaxel in paclitaxel-resistant MDA‑MB-231 cells was 74.400 nM, and that in paclitaxel-resistant SUM159PT cells was 75.570 nM.

**Immunohistochemistry (IHC)**

In this study, IHC staining was carried out in 183 cancer tissues from patients with TNBC and in mouse tumors. Briefly, formalin fixed paraffin-embedded (FFPE) specimens were cut into 4 μm thick slices and baked at 65 °C for 30 min. The slices were then deparaffinized using xylenes and rehydrated. Thereafter, the targeted antigens were retrieved using the microwave antigen retrieval method. Slices were treated with 3% H_2_O_2_ to quench the endogenous peroxidase activity, followed by incubation with 1% bovine serum albumin to block nonspecific binding. The slices were then incubated with the indicated primary antibodies at 4 °C overnight. Next day, the slices were treated with the indicated biotinylated secondary antibodies, followed by incubation with streptavidin-horseradish peroxidase complex (Zsbio, Beijing, China). Finally, the sections were immersed in 3-amino-9-ethyl carbazole and counterstained with 10% Mayer's hematoxylin, dehydrated, and mounted in Crystal Mount. The primary antibodies used for IHC staining included anti-ALYREF, and anti-RNF31 antibodies. For negative controls, the primary antibody was replaced with normal rabbit serum at 4 °C overnight preceding the immunohistochemical staining procedure.

**Plasmids, retroviral infection, and establishment of stable cell lines**

Stable cell lines were generated from cell pools via virus infection. Human *ALYREF*, *RNF31*, and *IPO13* coding sequences were amplified using PCR and subcloned into a pLVX‑IRES‑hygro vector for overexpression. RNF31 mutants were constructed based on the RNF31-wild-type (wt) and tagged with the MYC peptide sequence.

Small interfering RNA (siRNA) knockdown of endogenous *ALYREF*, *RNF31*, *KPNB1*, *KPNA2*, *IPO13*, *CBLL1*, *MKRN1*, and *MKRN2* were mediated via siRNA duplexes targeting the indicated mRNA sequences.

To knockdown endogenous *RNF31,* or *IPO13*, two short hairpin RNA (shRNA) oligonucleotides were cloned into the pSuper-neo vector, respectively. The oligonucleotide sequences of the shRNAs are provided below.

Briefly, the viral vectors were co-transfected with packaging plasmid (pMD2G, psPAX2 for pLVX-IRES-hygro and pIK for pSuper-neo) into HEK293FT cells. The supernatant containing the viruses was collected and viral infections were carried out serially for 3 days. Stable cell lines were selected using 25 μg/ml hygromycin for 6 days, or 250 μg/ml neomycin for 10 days, respectively. The efficiency of gene overexpression or knockdown was examined using qRT-PCR and western blotting.

**Protein immunoprecipitation (IP) assays**

Cell lysates were prepared from the indicated cells using lysis buffer (150 mM NaCl, 10 mM HEPES, pH 7.4, 1% NP-40). Lysates were then incubated with indicated antibodies and protein G-conjugated agarose (Millipore, Billerica, MA, USA) overnight at 4 °C. Beads containing affinity-bound proteins were washed six times using wash buffer (150 mM NaCl, 10 mM HEPES, pH 7.4, 0.1% NP-40), followed by elution using Laemmli Sample Buffer (Sigma-Aldrich, St. Louis, MO, USA). The eluates were subjected to mass spectrometry (MS) or western blotting analysis. Information on peptides and counts for ALYREF-binding proteins analyzed by IP/MS assays is provided in **Supplementary Table 6**.

**Cell subcellular fractionation**

Nuclear and cytoplasmic fractions of MDA-MB-231 or SUM159PT cells were prepared using a Minute™ Nuclear and Cytoplasmic Extraction Reagents Kit (Invent Biotechnologies, Plymouth, MN, USA; SC-003) according to the manufacturer’s protocol. Indicated cells were pre-treated with Thiolutin (1 μM) or paclitaxel (10 nM) for 48 h to impact ALYREF nuclear translocation, while DMSO treatment was used as a negative control. Thereafter, the isolated fractions were processed for western blotting analysis as stated.

**Cell viability assay**

The indicated cells were plated on 96-well plates (4000 cells per well). After adding Cell counting kit-8 reagent (DOJINDO, Kumamoto, Japan) to the wells for 2 h at 37 °C in the dark, the OD at 450 nm was measured using a microplate reader (EPOCH2, BioTek, Winooski, VT, USA). Each cell line was set up in three replicate wells, and the experiment was repeated three times.

**Colony formation assay**

A colony formation assay was performed to determine cells’ colony formation ability. The indicated cells (1 × 10^3^) were plated in 6-well plates and administered with the indicated treatments for 48 h. The cell medium was refreshed and culture was continued for 14 days. Thereafter, the surviving colonies were fixed, stained with crystal violet, and counted.

**Terminal deoxynucleotidyl transferase nick-end labeling (TUNEL) assay**

The cell apoptosis rate was measured using a DeadEnd Fluorometric TUNEL System (KeyGEN Biotech, Nanjing, China), following the manufacturer’s instructions. Briefly, paraffin embedded sections were baked at 65 °C for 60 min, washed three times in a xylene bath, washed in turn in absolute ethanol, 95% ethanol, and 70% ethanol, and three times with phosphate-buffered saline (PBS). Each section was incubated with proteinase K at 37 °C for 30 min and washed three times with PBS. Subsequently, the sections were incubated with equilibration buffer (containing Biotin-11-dUTP and TdT Enzyme) at 37 ℃ for 1 h in the dark, followed by washing with PBS three times. Next, the samples were incubated with Streptavidin-Fluorescein at 37 ℃ for 30 min in the dark, followed by washing with PBS three times. 4′,6-diamidino-2-phenylindole (DAPI) was added to stain the nuclei. Finally, the experimental results were collected under a fluorescence microscope with a 450 to 500 nm excitation wavelength and a 515 to 565 nm emission wavelength. The proportion of TUNEL positive cells was quantified from five random fields.

**RNA immunoprecipitation assays**

RNA immunoprecipitation (RIP) assays were used to detect interactions between proteins and mRNAs in TNBC cells. Briefly, cells were starved for 24 hours and then stimulated with a standard culture medium containing 10% fetal bovine serum (FBS) to initiate robust gene transcription. For the Thiolutin group, Thiolutin (1 μM) was used to pre-treat the cells for 48 hours before starvation. Cells were harvested 8 hours later and lysed by using lysis buffer (20 mmol/L Tris-Cl, pH 8.0, 10 mmol/L NaCl, 1 mmol/L EDTA, 0.5% NP-40) supplemented with RNasin (Promega, Madison, WI, USA). Lysates were then incubated with anti-ALYREF or anti-hMTR4 antibodies, and then washed five times with lysis buffer. The retrieved pellets were then subjected to qRT-PCR analysis using specific primers for *TUBB3*, *STMN1*, and *TAU*.

**Immunofluorescence (IF) assay**

After fixation with 4% paraformaldehyde, permeabilization in 0.2% Triton X-100 (PBS), and blocking nonspecific binding with 1% bovine serum albumin, cells were incubated with the indicated antibodies overnight at 4 °C. Next day, the cells were returned to room temperature for 30 min, followed by incubation with fluorescein-conjugated secondary antibodies (CST, Danvers, MA, USA; diluted at 1:500) for 1 h at room temperature. The nuclei were stained with DAPI, and images were visualized under a Nikon Eclipse Ti2-E microscope. The subcellular proportion of ALYREF was quantified from five random fields using Image J software (NIH, Bethesda, MD, USA), and each experiment was repeated in three times.

**Quantitative real-time reverse transcription PCR**

Isolated mRNA was converted to cDNA using the ReverTra Ace® (Toyobo, Osaka, Japan; TRT-101). The quantitative real-time PCR reactions were carried out by employing the cDNA as the template and SYBR Green PCR master mix (Toyobo, QPK201). Primers for *ALYREF*, *RNF31*, *IPO13*, *TUBB3*, *STMN1*, and *TAU* are provided below.

**Western blotting analysis.**

Western blotting analyses were performed according to a standard protocol using primary antibodies, with histone H3 was used as the loading control for the nuclear fraction, and β‑Actin was used as the loading control for whole cell lysate.

**Primers and oligonucleotides**

**Primers for qRT-PCR**

*ALYREF*, forward primer, 5'-GGAGTCTCAGACGCCGATATTC-3';

reverse primer, 5'-GCATCTGCCTTCCGCTCAAAGT-3'.

*RNF31*, forward primer, 5'-ACTGGCGTGGTGTCAAGTTTA-3';

reverse primer, 5'-CGGGGAAGCTCAACCCATC -3'.

*IPO13*, forward primer, 5'-ATGCCCAGAGGTACGTGAAC-3';

reverse primer, 5'-CACAGCGATGCGACAGATG-3'.

*TUBB3*, forward primer, 5'- GGCCAAGGGTCACTACACG -3';

reverse primer, 5'- GCAGTCGCAGTTTTCACACTC -3'.

*STMN1*, forward primer, 5'-AGAACCGAGAGGCACAAATGGC-3';

reverse primer, 5'-TCTCGTCAGCAGGGTCTTTGGA-3'.

*TAU*, forward primer, 5'-CCAGTCCAAGTGTGGCTCAAAG-3';

reverse primer, 5'-GCCTAATGAGCCACACTTGGAG-3'.

*ACTB*, forward primer, 5’-GCAAAGACCTGTACGCCAACA-3’;

reverse primer, 5’-TGCATCCTGTCGGCAATG-3’.

**Gene silencing oligonucleotides**

siALYREF#1, target sequence: 5'-CAGACGCCGATATTCAGGAACTCTT-3'.

siALYREF#2, target sequence: 5'- GCCGATATTCAGGAACTCTTTGC-3'.

siRNF31, target sequence: 5'-GCCCAGAGTCAAGTCTGGTACTGTA-3'.

siCBLL1, target sequence: 5'-AGGATGATACACCAGTTCAT-3'.

siMKRN1, target sequence: 5'-CATCCAATGGATGCTGCCCAGAGAT-3'.

siMKRN2, target sequence: 5'-CGGGAAGGAAGTCAGTGCCTATTCT-3'.

siKPNA2, target sequence: 5'-CCAAGCTACTCAAGCTGCCAGGAAA-3'.

siKPNB1, target sequence: 5'- CGGTTATATTTGCCAAGATATAG -3'.

siIPO13, target sequence: 5'-CAGGATGATATTCTATCCTTTGA-3'.

shRNF31#1, target sequence: 5'-GCTGCAGCTTTCAGAATTTGA-3'.

shRNF31#2, target sequence: 5'- GCACACTACAAAGAGTATCTT-3'.

shIPO13, target sequence: 5'- GACACTGTCATGTTCACCATT -3'.

**Antibodies used in the present study**

| **Name** | **Catalog Number** | **Company** | **Concentration** | **Source** |
| --- | --- | --- | --- | --- |
| **IHC** | | |  |  |
| **Anti-ALYREF** | **HPA019799** | **Sigma-Aldrich** | 1:200 | **Rabbit** |
| **Anti-RNF31** | **HPA048745** | **Sigma-Aldrich** | 1:200 | **Rabbit** |
| **WB** | | |  |  |
| **Anti-ALYREF** | **HPA019799** | **Sigma-Aldrich** | 1:1000 | **Rabbit** |
| **Anti-RNF31** | **#99633** | **CST** | 1:1000 | **Rabbit** |
| **Anti-KPNB1** | **#51186** | **CST** | 1:1000 | **Rabbit** |
| **Anti-KPNA2** | **#14372** | **CST** | 1:1000 | **Rabbit** |
| **Anti-IPO13** | **ab95993** | **Abcam** | 1:1000 | **Rabbit** |
| **Anti-TUBB3** | **#5568** | **CST** | 1:1000 | **Rabbit** |
| **Anti-STMN1** | **#13655** | **CST** | 1:1000 | **Rabbit** |
| **Anti-TAU** | **#46687** | **CST** | 1:1000 | **Rabbit** |
| **Anti-Caspase 3** | **#9662** | **CST** | 1:1000 | **Rabbit** |
| **Anti-Cleaved-caspase 3** | **#9661** | **CST** | 1:1000 | **Rabbit** |
| **Anti-Linear ubiquitin** | **MABS451** | **Sigma-Aldrich** | 1:1000 | **Mouse** |
| **Anti-β-Actin** | **#4970** | **CST** | 1:1000 | **Rabbit** |
| **Anti-Histone H3** | **#4499** | **CST** | 1:2000 | **Rabbit** |
| **Anti-FLAG** | **#14793** | **CST** | 1:1000 | **Rabbit** |
| **Anti-MYC** | **#18583** | **CST** | 1:1000 | **Rabbit** |
| **Anti-HIS** | **#12698** | **CST** | 1:1000 | **Rabbit** |
| **Anti-NXF1** | **#12735** | **CST** | 1:1000 | **Rabbit** |
| **Anti-NXT1** | **ab168481** | **Abcam** | 1:1000 | **Mouse** |
| **Anti-UAP56** | **#47258** | **CST** | 1:1000 | **Rabbit** |

| **Name** | **Catalog Number** | **Company** | **Concentration** | **Source** |
| --- | --- | --- | --- | --- |
| **WB** | | | | |
| **Anti-THOC5** | **ab137051** | **Abcam** | 1:2000 | **Rabbit** |
| **Anti-Ran** | **#4462** | **CST** | 1:1000 | **Rabbit** |
| **Anti-RanBP1** | **#8780** | **CST** | 1:1000 | **Rabbit** |
| **IF** |  |  |  |  |
| **Anti-ALYREF** | **HPA019799** | **Sigma-Aldrich** | 1:200 | **Rabbit** |
| **IP/RIP** |  |  |  |  |
| **Anti-ALYREF** | **#12655** | **CST** | 1:50 | **Rabbit** |
| **Anti-RNF31** | **#99633** | **CST** | 1:50 | **Rabbit** |
| **Anti-IPO13** | **ab95993** | **Abcam** | 1:50 | **Rabbit** |
| **Anti-hMRT4** | **ab70551** | **ABCAM** | 1:50 | **Rabbit** |
| **Anti-**FLAG | **#14793** | **CST** | 1:50 | **Rabbit** |
